# Boosting LPMO-driven lignocellulose degradation by polyphenol oxidase-activated lignin building blocks

**DOI:** 10.1186/s13068-017-0810-4

**Published:** 2017-05-10

**Authors:** Matthias Frommhagen, Sumanth Kumar Mutte, Adrie H. Westphal, Martijn J. Koetsier, Sandra W. A. Hinz, Jaap Visser, Jean-Paul Vincken, Dolf Weijers, Willem J. H. van Berkel, Harry Gruppen, Mirjam A. Kabel

**Affiliations:** 1 Laboratory of Food Chemistry, Wageningen University & Research, Bornse Weilanden 9, 6708 WG Wageningen, The Netherlands; 2 Laboratory of Biochemistry, Wageningen University & Research, Stippeneng 4, 6708 WE Wageningen, The Netherlands; 3 DuPont Industrial Biosciences, Nieuwe Kanaal 7-S, 6709 PA Wageningen, The Netherlands; 4 Fungal Genetics & Technology Consultancy, P.O. Box 39b, 6700 AJ Wageningen, The Netherlands

**Keywords:** LPMO, *Myceliophthora thermophila* C1, Phenols, Tyrosinase, Catechol oxidase, Polyphenol oxidase, PPO, *Agaricus bisporus*, Ascomycota, Basidiomycota, Lignocellulose

## Abstract

**Background:**

Many fungi boost the deconstruction of lignocellulosic plant biomass via oxidation using lytic polysaccharide monooxygenases (LPMOs). The application of LPMOs is expected to contribute to ecologically friendly conversion of biomass into fuels and chemicals. Moreover, applications of LPMO-modified cellulose-based products may be envisaged within the food or material industry.

**Results:**

Here, we show an up to 75-fold improvement in LPMO-driven cellulose degradation using polyphenol oxidase-activated lignin building blocks. This concerted enzymatic process involves the initial conversion of monophenols into diphenols by the polyphenol oxidase *Mt*PPO7 from *Myceliophthora thermophila* C1 and the subsequent oxidation of cellulose by *Mt*LPMO9B. Interestingly, *Mt*PPO7 shows preference towards lignin-derived methoxylated monophenols. Sequence analysis of genomes of 336 Ascomycota and 208 Basidiomycota reveals a high correlation between *Mt*PPO7 and AA9 LPMO genes.

**Conclusions:**

The activity towards methoxylated phenolic compounds distinguishes *Mt*PPO7 from well-known PPOs, such as tyrosinases, and ensures that *Mt*PPO7 is an excellent redox partner of LPMOs. The correlation between *Mt*PPO7 and AA9 LPMO genes is indicative for the importance of the coupled action of different monooxygenases in the concerted degradation of lignocellulosic biomass. These results will contribute to a better understanding in both lignin deconstruction and enzymatic lignocellulose oxidation and potentially improve the exploration of eco-friendly routes for biomass utilization in a circular economy.

**Electronic supplementary material:**

The online version of this article (doi:10.1186/s13068-017-0810-4) contains supplementary material, which is available to authorized users.

## Background

Fungal carbohydrate converting enzymes are considered important for eco-friendly application in plant biomass degradation. The resulting carbohydrates are sources for the production of biochemicals or biofuels and new enzymatically modified cellulose-based products are envisaged for the future.

Next to carbohydrates, phenolic compounds are also major components of lignocellulosic plant biomass. Phenolic compounds are present either as free molecules or in conjugated form as part of lignin or bound to carbohydrates. Lignin is one of the major constituents of (secondary) plant cell walls, together with the polysaccharides cellulose and hemicellulose. Lignin is composed of the three aromatic monolignol units: coniferyl, sinapyl, and *para*-coumaryl alcohol. Cellulose consists of β-(1→4)-linked glucosyl chains that interact with each other via hydrogen bonds and van der Waals forces, which results in the formation of crystalline cellulose fibrils. Unlike cellulose, hemicellulose is a heteropolymer varying in its monosaccharide composition and linkages between the monosaccharides. Examples are xylan, mannan, or β-(1→3, 1→4)-linked β-glucan. Hemicellulose interacts with lignin through ester and ether linkages, thereby forming a network that embeds the cellulose microfibrils [1, 2].

Recent studies focused on the function of lytic polysaccharide monooxygenases (LPMOs) have confirmed that these enzymes drive the oxidative degradation of cellulose, and they are considered important for the enzymatic degradation of plant biomass [3]. LPMOs are classified based on their sequence in the Carbohydrate Active enzyme (CAZy; [4]) database as auxiliary activity (AA) families AA9, AA10, AA11, and AA13. In brief, LPMOs have been reported to oxidize β-(1→4)-linked glucan chains at either the C1- or C4-carbon position or both, resulting in the cleavage of glucan chains [3, 5, 6]. Other LPMOs of these AA families have been described to oxidize the (1→4)-linkage of chitin, xylan, hemicellulose, such as xyloglucan and glucomannan, soluble cellodextrins, and starch [7–11].

In order to oxidize polysaccharides, LPMOs demand electrons to activate molecular oxygen in their copper-containing active site [3, 5, 6]. The electrons can be donated by reducing agents, like low molecular weight compounds (ascorbic acid, gallic acid) or the macromolecule lignin [12–14]. Other ways of providing LPMOs with electrons have been reported, such as flavocytochrome-dependent cellobiose dehydrogenases (CDHs), light-induced pigments, light-driven chemical oxidation of water, or diphenol-regenerating GMC (glucose-methanol-choline-oxidase/dehydrogenase)-oxidoreductases [5, 12, 15, 16]. The mechanistic understanding of electron donation systems is highly relevant in order to enable optimization of LPMO activity and, thereby, plant biomass degradation.

Phenolic compounds, including small molecular weight compounds that serve as lignin building blocks and lignin, are intrinsically present in plant biomass and are natural electron donors for LPMO activity. However, monophenols are not optimal electron donors for LPMO activity, because of their relatively high redox potential [12]. Compounds with a 1,2-benzenediol or a 1,2,3-benzenetriol moiety have, compared to monophenols, a lower redox potential [13]. Their low redox potential enables them to reduce the copper ion in the active site of LPMOs and enhance the LPMO activity [12].

The enzymatic oxidation of phenolic compounds is a well-known reaction in many natural environments. For example, it causes browning of food products, contributes to taste in tea fermentation, and plays a role in plant biomass decomposition [17–19]. These oxidative systems involve the activity of laccases (EC.1.10.3.2), peroxidases (EC.1.11.1), tyrosinases (EC.1.14.18.1), and catechol oxidases (EC 1.10.3.1) [18–20]. In particular tyrosinases, also often referred to as polyphenol oxidases, are of interest due to their ability to hydroxylate phenolic compounds [21]. This so-called monophenolase activity typically involves the *ortho*-hydroxylation of monophenols into *ortho*-diphenols, compounds that comprise a 1,2-benzenediol moiety. Tyrosinases also exhibit diphenolase activity, which is characterized by the oxidation of these *ortho*-diphenols into *ortho*-quinones [22]. With respect to biomass degradation, the diphenolase activity of polyphenol oxidases is not conductive to polysaccharides oxidation since *ortho*-quinones cannot be utilized by LPMOs [12]. Based on their monophenolase activity, tyrosinases have been shown to use non-methoxylated monophenols as substrates, rather than using methoxylated monophenols, that are the predominant structural units of lignin [23].

In this study, we investigated if polyphenol oxidases, in particular tyrosinases, can enhance LPMO activity. Therefore, the tyrosinase *Mt*PPO7 from *Myceliophthora thermophila* C1 was used, which showed activity towards phenolic compounds and was obtained from DuPont Industrial Biosciences. In addition, we used the commercially available tyrosinase *Ab*PPO from the white button mushroom *Agaricus bisporus*. Both tyrosinases were each incubated with the previously described *Mt*LPMO9B in the presence of various plant phenolic compounds and cellulose [13, 24]. We found that *Mt*PPO7 is highly active towards methoxylated monophenols, including monomeric lignin building blocks. This activity can strongly boost the release of oxidized gluco-oligosaccharides as catalyzed by *Mt*LPMO9B and thereby the degradation of cellulose. In addition, we found a strong correlation between genes encoding *Mt*PPO like proteins and AA9 LPMOs in fungal genomes of 336 Ascomycota and 208 Basidiomycota.

## Results

### *Mt*PPO7 addition improves cellulose oxidation by *Mt*LPMO9B

**Fig. 1:**
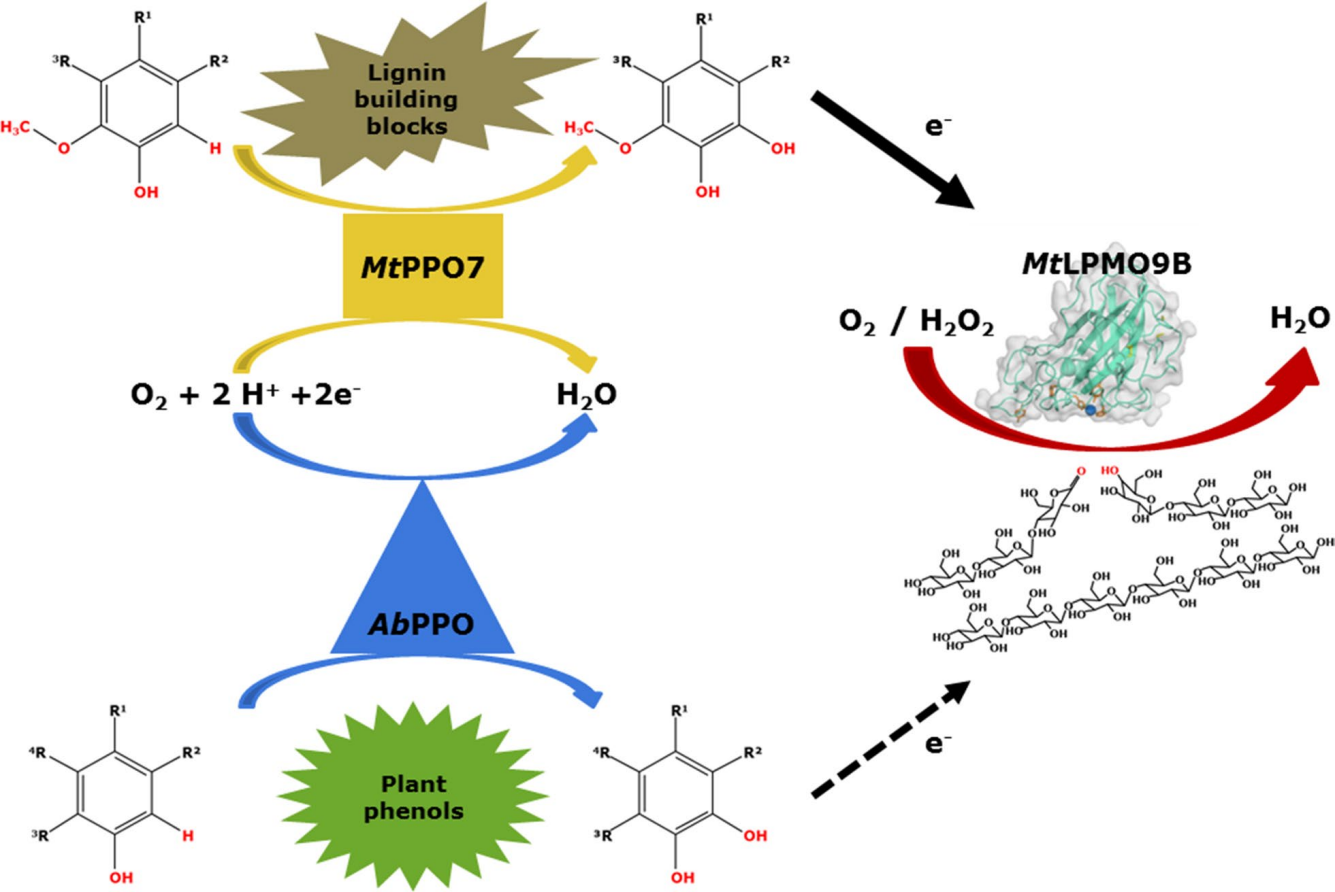
Schematic presentation of the concerted action of polyphenol oxidases and LPMOs. Monophenols with a 1-hydroxy, 2-methoxy moiety are hydroxylated by *Mt*PPO7 from *M. thermophila* C1. The resulting methoxylated catechols are excellent electron donors for *Mt*LPMO9B activity. In comparison to *Mt*PPO7, the mushroom tyrosinase *Ab*PPO is able to convert non-methoxylated monophenols into compounds comprising a 1,2-benzenediol moiety. The released compounds comprising a 1,2-benzenediol moiety are able to donate electrons for *Mt*LPMO9B activity. However, *Ab*PPO exhibits a strong diphenolase activity which reduces the available amount of these compounds comprising a 1,2-benzenediol moiety for *Mt*LPMO9B due to further oxidation of these compounds into *ortho*-quinones (indicated by *dashed arrow*)

Our previous results have shown that reducing agents with a 1,2-benzenediol or 1,2,3-benzenetriol moiety gave the highest release of non-oxidized and C1-oxidized gluco-oligosaccharides from regenerated amorphous cellulose (RAC) incubated with three *Mt*LPMOs compared to the incubation with compounds comprising only a single hydroxyl group [13]. Hence, in the current research we hypothesized that *Mt*LPMO activity can benefit from enzymes which have hydroxylating capacity, such as polyphenol oxidases (Fig. 1) [21]. We choose *Mt*PPO7, which originates like the *Mt*LPMO9B employed here, from the thermophilic filamentous fungus *Myceliophthora thermophila* C1. As a reference, the well-studied tyrosinase *Ab*PPO from the edible mushroom *Agaricus bisporus* was used [19, 25–28].

**Fig. 2:**
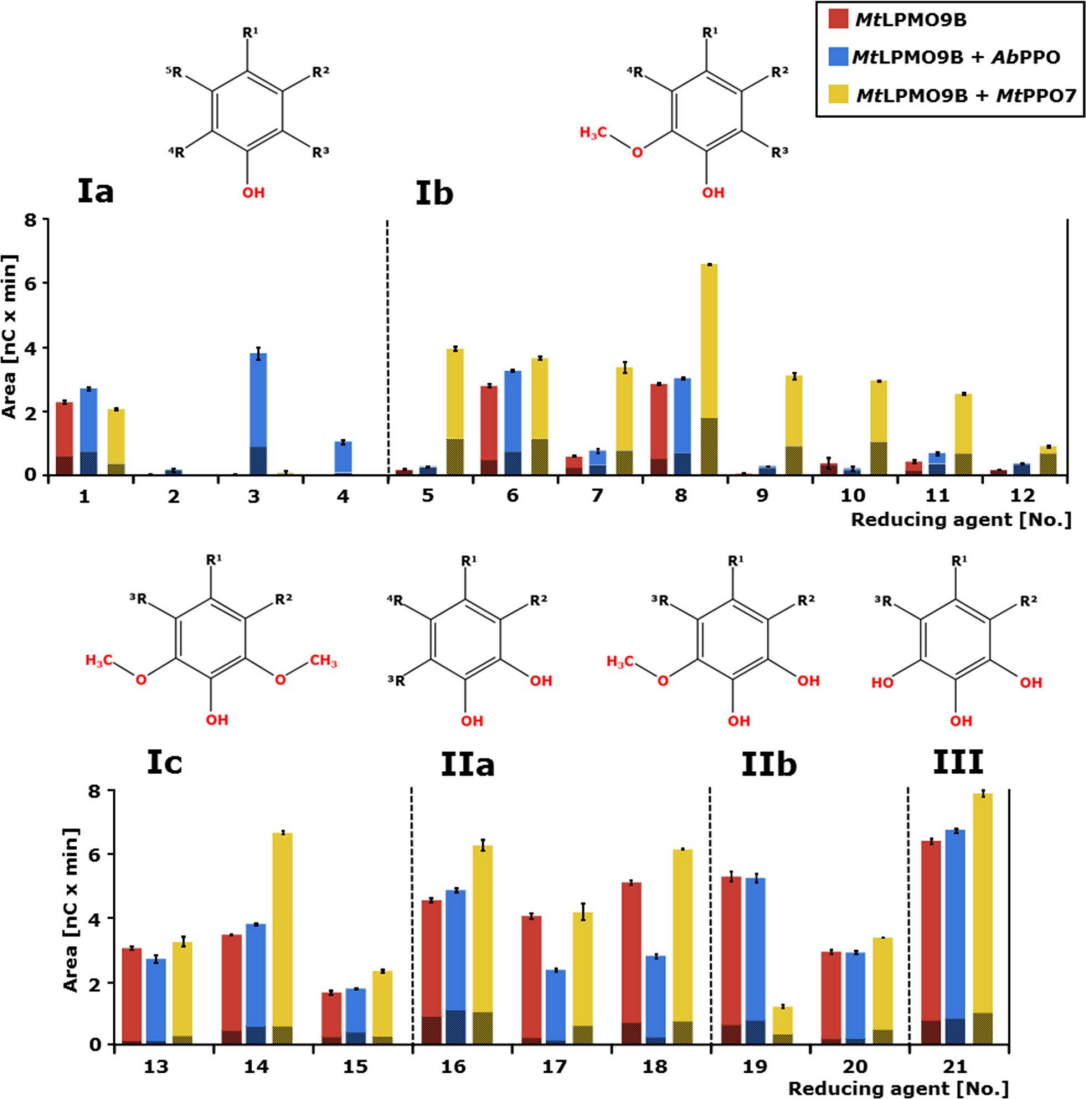
RAC incubated with *Mt*LPMO9B with or without *Mt*PPO7 or *Ab*PPO addition in the presence of 21 reducing agents. The* numbers* are total sums of the integrated peak areas of released non-oxidized (*shaded red*, *blue*, and *yellow*) and C1-oxidized (*red*, *blue,* and *yellow*) gluco-oligosaccharides after incubation of regenerated amorphous cellulose (RAC; 1.5 mg mL^−1^) with *Mt*LPMO9B only (*red bars*) (5.0 μg mL^−1^), *Mt*LPMO9B together with *Ab*PPO (*blue bars*, 2.5 µL mL^−1^), or *Mt*LPMO9B together with *Mt*PPO7 (*yellow bars*) (5.0 μg mL^−1^) based on HPAEC. The reducing agents (2 mM) are numbered (*X-axis*) and specified in Table 1. Vertical* dotted lines* separate the reducing agents into groups (I, II, II) and subgroups (a, b, c). Group I, monophenols (**Ia**), compounds with a 1-hydroxy,2-methoxy moiety (**Ib**) or a 1-hydroxy-2,6-dimethoxy moiety (**Ic**). Group II, compounds with 1,2-benzenediols moiety (**IIa**) and compounds with a 1,2-dihydroxy-3-methoxy moiety (**IIb**). Group III, reducing agents with a 1,2,3-benzenetriol moiety (**III**). All incubations were performed in duplicate and the standard deviations are represented by *error bars*. See “Methods” for details

The activity of *Mt*LPMO9B towards RAC, with and without addition of *Mt*PPO7, was determined using 21 phenolic compounds as potential electron donors. Many of these phenolic compounds are methoxylated as these are the predominant structural units of lignin. Results are presented in Table 1 and Fig. 2. The 21 different phenolic compounds used were classified into three groups and further divided into subgroups, as previously described [13]. In short, group I represents compounds comprising one hydroxyl group, group II compounds comprising a 1,2-benzenediol moiety, and group III compounds comprising a 1,2,3-benzenetriol moiety (Fig. 2; Table 1).

**Table 1:** RAC incubated with *Mt*LPMO9B only and with *Ab*PPO or *M*tPPO7 addition in the presence of 21 reducing agents

Gr.	Sub-group	No.	Reducing agent	Activity [%] *Mt*LPMO9B^a^	Activity [%] *Mt*LPMO9B + *Ab*PPO^b^	Activity [%] *Mt*LPMO9B + *Mt*PPO7^b^
I	a	1	4-Hydroxybenzoic acid	100	119	90
		2	*Ortho*-cresol	100	*778*	2
		3	*Para*-coumaric acid	100	*9938*	*2851*
		4	Phenol	0^c^	*>0* ^c^	0^c^
I	b	5	3-Hydroxy-4-methoxycinnamic acid	100	*151*	*7558*
		6	4-Hydroxy-3-methoxyphenylacetone	100	117	131
		7	Coniferyl aldehyde	100	128	*562*
		8	Ferulic acid	100	107	*231*
		9	Guaiacol	100	*461*	*5143*
		10	Hesperidin	100	56	*771*
		11	Homovanillic acid	100	*156*	*580*
		12	Vanillic acid	100	*196*	*495*
I	c	13	4-Allyl-2,4-methoxyphenol	100	89	108
		14	Sinapic acid	100	110	*195*
		15	Syringic acid	100	107	142
II	a	16	3,4-Dihydroxybenzoic acid	100	99	139
		17	3-Methylcatechol	100	58	104
		18	caffeic acid	100	55	122
II	b	19	3,4-Dihydroxy-5-methoxybenzoic acid	100	99	22
		20	3,4-Dihydroxy-5-methoxycinnamic acid	100	99	116
III		21	Gallic acid	100	105	124

Division based on functional groups of reducing agents (Fig. 2)

^a^Total release of non-oxidized and C1-oxidized gluco-oligosaccharides from RAC incubated with *Mt*LPMO9B set to 100%

^b^Increased (italics ≥50% increase) or decreased percentage of released non-oxidized and C1-oxidized gluco-oligosaccharides from RAC incubated with *Mt*LPMO9B with addition of either *Ab*PPO or *Mt*PPO7 compared to the release of non-oxidized and C1-oxidized gluco-oligosaccharides from RAC incubated with *Mt*LPMO9B alone. Sum of areas of released non-oxidized and C1-oxidized gluco-oligosaccharides are shown in Fig. 2. See “Methods” for more information

^c^No reference due to absent activity of *Mt*LPMO9B towards RAC in the presence of phenol (Fig. 2)

As expected, from our previous research [13], monophenols (group Ia) are less efficient electron donors for *Mt*LPMO9B, with the exception of 4-hydroxybenzoic acid (no. 1). The low *Mt*LPMO9B activity towards RAC resulted in a very small amount of released non-oxidized and C1-oxidized gluco-oligosaccharides (Fig. 2; Table 1; Additional file 1: Figure S1). The addition of *Mt*PPO7 to RAC incubated with *Mt*LPMO9B did not increase the electron donor capacities of this group Ia compounds, except for *para*-coumaric acid (no. 3). In contrast, the addition of *Mt*PPO7 to RAC incubated with *Mt*LPMO9B in the presence of methoxylated monophenols (group Ib, compounds with a 1-hydroxy, 2-methoxy moiety) led in all cases to a high increase (up to ±75 times, no. 5) in the release of non-oxidized and C1-oxidized gluco-oligosaccharides (Fig. 2; Additional file 1: Figure S1). Similarly, the oxidative cleavage of RAC incubated with *Mt*LPMO9B increased, when *Mt*PPO7 was added to group Ic compounds, which comprise a 1-hydroxy-2,6-dimethoxy moiety. For group IIa, IIb, and III phenolic compounds, the addition of *Mt*PPO7 to incubations of RAC with *Mt*LPMO9B led to a moderate increase of non-oxidized and C1-oxidized gluco-oligosaccharides (4–39%), with one exception (no. 19) (Fig. 2; Table 1).

Incubations performed with *Ab*PPO showed deviating results from corresponding experiments with *Mt*PPO7. First, unlike *Mt*PPO7, addition of *Ab*PPO to non-methoxylated monophenols (group Ia) resulted in increased levels of non-oxidized and C1-oxidized oligosaccharides during the incubation of RAC with *Mt*LPMO9B. This different activity was expected on the basis of the known hydroxylating capacity of *Ab*PPO towards non-methoxylated monophenolic compounds. Especially, the addition of *Ab*PPO in the presence of *para*-coumaric acid (no. 3) increased *Mt*LPMO9B-catalyzed degradation of RAC compared to the incubation without *Ab*PPO (Fig. 2; Table 1). In contrast to *Mt*PPO7, *Ab*PPO addition to group Ib, Ic, II, and III compounds improved the *Mt*LPMO9B-catalyzed RAC degradation relatively moderate (5–361%, Table 1) or even decreased the RAC degradation by 1–44% (Fig. 2; Table 1).

### *Mt*LPMO9B-mediated cellulose oxidation in the presence of *Mt*PPO7 using time-dependent incubations

**Fig. 3:**
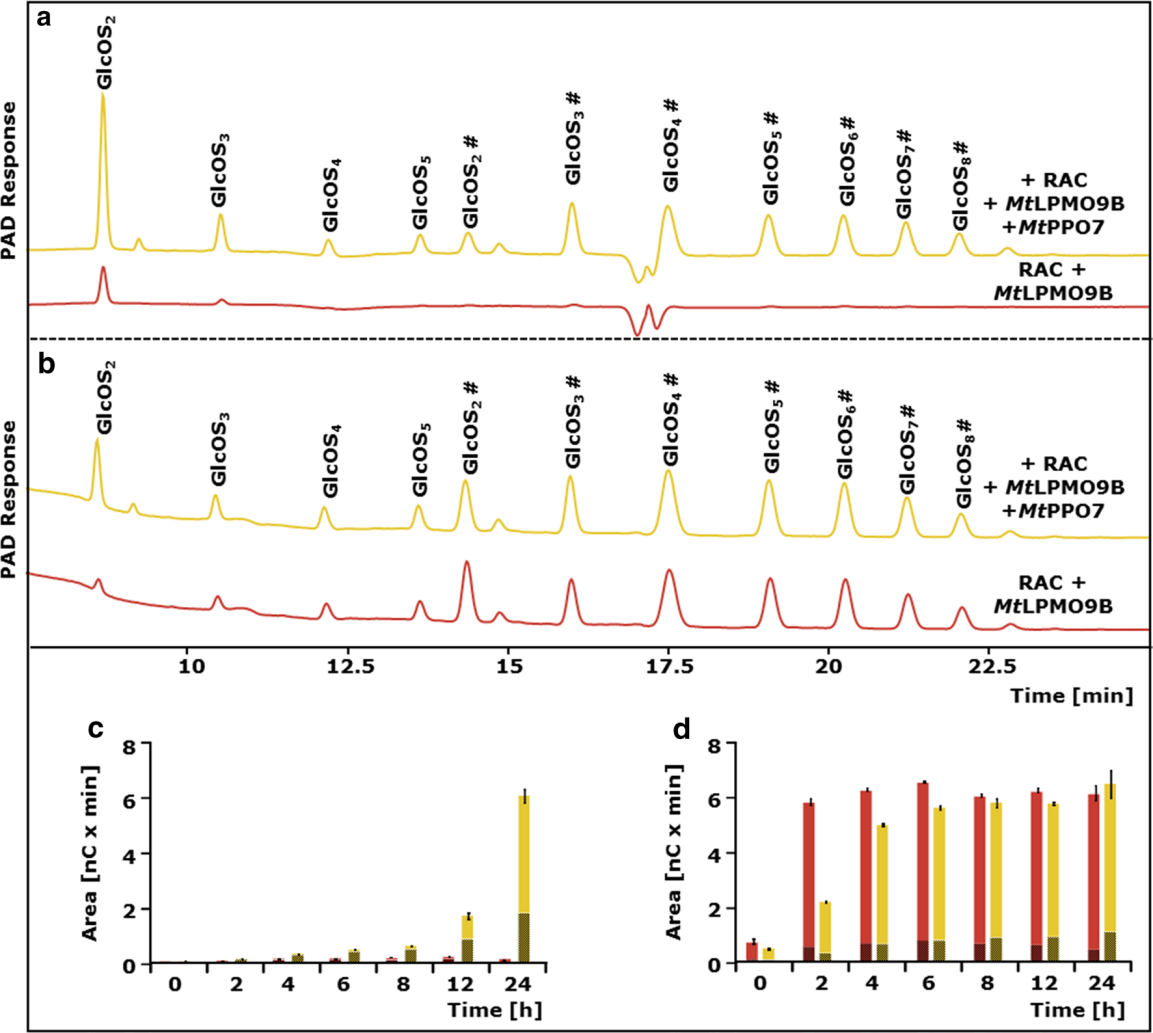
Activity of *Mt*LPMO9B towards amorphous cellulose in the presence and absence of *Mt*PPO7. HPAEC elution pattern of regenerated amorphous cellulose (RAC; 1.5 mg mL^−1^) incubated with *Mt*LPMO9B (5.0 μg mL^−1^) with (*yellow*) and without (*red*) *Mt*PPO7 (5.0 μg mL^−1^) addition in the presence of **a** guaiacol (no. 9 specified in Table 1, 2 mM) and **b** 3-methylcatechol (no. 17 specified in Table 1, 2 mM) after 24 h. The incubation of RAC with *Mt*LPMO9B results in the formation of non-oxidized gluco-oligosaccharides (GlcOS_n_) and C1-oxidized gluco-oligosaccharides (GlcOS_n_^#^). Based on HPAEC, integrated peak areas are shown as the total sum of released non-oxidized (*shaded red* and *yellow*) and C1-oxidized (*red* and *yellow*) gluco-oligosaccharides after incubation of RAC (1.5 mg mL^−1^) with *Mt*LPMO9B only (*red bars*, 5.0 μg mL^−1^) and *Mt*LPMO9B together with *Mt*PPO7 (*yellow bars*, 5.0 μg mL^−1^) in the presence of **c** guaiacol and **d** 3-methylcatechol. All incubations were performed in duplicate, and the standard deviations are presented as *error bars*. See “Methods” for data analysis and details

The ability of *Mt*PPO7 to convert methoxylated phenolic compounds and to increase the oxidative degradation of RAC by *Mt*LPMO9B was further investigated using time-dependent incubations (24 h). These incubations were performed in the presence of two methoxylated monophenols (guaiacol and ferulic acid, group Ib), as well as in the presence of a non-methoxylated *ortho*-diphenol (3-methylcatechol, group IIa) (Fig. 3; Additional file 2: Figure S2). After 4 h of incubation, a steady *Mt*PPO7-induced oxidation of guaiacol (no. 9) was observed (Additional file 3: Figure S3). At the same time, the amounts of released non-oxidized and C1-oxidized gluco-oligosaccharides by *Mt*LPMO9B from RAC also increased steadily (Fig. 3c). A similar trend for the release of non-oxidized and C1-oxidized gluco-oligosaccharides was shown for the incubation with ferulic acid (no. 8, Additional file 2: Figure S2). *Mt*PPO7 showed a relatively low efficiency towards 3-methylcatechol (no. 17) compared to guaiacol (Table 1; Additional file 3: Figure S3). As reported above, no significant increase of *Mt*LPMO9B-released non-oxidized and C1-oxidized gluco-oligosaccharides from RAC was observed when comparing incubations with and without *Mt*PPO7 (Fig. 3d). In presence of *Mt*PPO7 though, the initial rate (0–6 h) of released non-oxidized and C1-oxidized gluco-oligosaccharides was lower compared to the incubation without *Mt*PPO7 (Fig. 3d).

### Activity of *Mt*PPO7 towards methoxylated phenolic compounds

The most striking observation from the experiments described above is the conversion of methoxylated phenolic compounds by *Mt*PPO7 into products, which enhance the oxidative degradation of cellulose by *Mt*LPMO9B. The fate of these phenolic compounds upon *Mt*PPO7 incubation was further studied in the absence of *Mt*LPMO9B using UHPLC-UV-MS^n^.

**Fig. 4:**
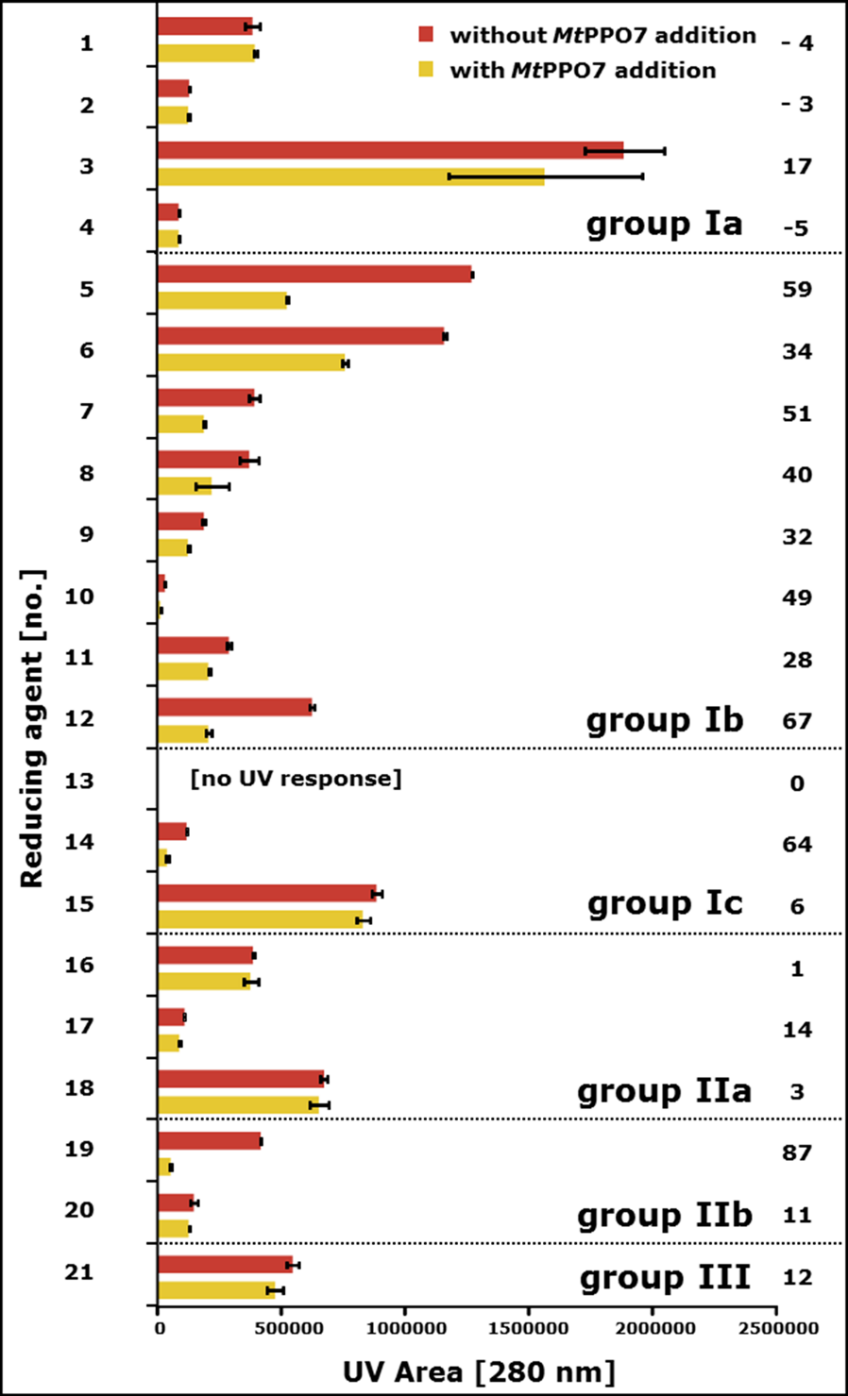
UV response areas of 21 reducing agents incubated in the presence and absence of *Mt*PPO7. The total sum is shown of integrated peak areas of 21 reducing agents (2 mM) with (*yellow bar*) and without (*red bar*) addition of *Mt*PPO7 (5.0 μg mL^−1^). The reducing agents are numbered (*bold on the left*) and specified in Table 1. Samples were incubated in a 50 mM potassium phosphate buffer (pH = 6.0) containing 2.5 µM copper(II)-chloride for 24 h at 50 °C and measured by UHPLC-UV (280 nm). *Bold numbers on the right* reducing agents conversion (%) by *Mt*PPO7, which is based on the difference of integrated peak areas (UV 280 nm) of the reducing agents incubated with *Mt*PPO7 compared to the incubation of reducing agents only. All incubations were performed in duplicate. See “Methods” for details

Based on UV measurements, non-methoxylated phenolic compounds of group Ia, group IIa, and group III remained constant in concentration during *Mt*PPO7 incubation, showing that *Mt*PPO7 has a low activity towards compounds comprising a 1,2-benzenediol moiety (Fig. 4; Additional file 4: Table S1). Different from *Mt*PPO7, in previous research *Ab*PPO has been reported to be highly active towards these types compounds [19, 24, 25].

*Mt*PPO7 was active towards *all* eight methoxylated monophenols (group Ib) tested. Based on UV, the concentrations of group Ib compounds decreased during *Mt*PPO7 incubation between 28 and 67% compared to the same compounds incubated without *Mt*PPO7 (Fig. 4). Three types of *Mt*PPO7 reactions were observed based on mass differences between substrate and products formed (Additional file 4: Table S1): (A) hydroxylation (*m*/*z* +16; e.g., no. 5 and 7), (B) decarboxylation (*m*/*z* −44, e.g., no. 8), and (C) demethylation (*m*/*z* −14, e.g., no. 11). Especially the hydroxylation of methoxylated phenolic compounds is a key reaction, since the products formed comprise a second hydroxyl group. The formed *ortho*-diphenols are known to be efficient electron donors for LPMOs. Decarboxylation and demethylation occurred either in the presence of *Mt*PPO7 or as a result of polymerization reactions in both the presence and absence of *Mt*PPO7. Several group Ib compounds spontaneously formed dimers in the absence of *Mt*PPO7 (no. 8 or no. 11). These dimers were almost absent when group Ib compounds were incubated with *Mt*PPO7 (Additional file 4: Table S1). Similar to group Ib compounds, the concentrations of compounds comprising a 1-hydroxyl-2,6-dimethoxy moiety (group Ic) incubated with *Mt*PPO7 decreased, based on UV, between 6% and 64% compared to the incubation without *Mt*PPO7 (Fig. 4; Additional file 4: Table S1). Again, masses indicating decarboxylation (−44 Da) and demethylation (−14 Da) reactions were formed. *Mt*PPO7 was also able to convert group IIb compounds (up to 87% substrate conversion, no. 20) that comprise a 1,2-dihydroxy-3-methoxy moiety (Fig. 4). The reactions observed were decarboxylation (*m*/*z* −44, e.g., no. 19) and dimerization (e.g., no. 20) of group IIb compounds based on masses formed (Additional file 4: Table S1).

In general, most phenolic compounds (such as no. 17 and no. 21) that were incubated with *Mt*PPO7 formed insoluble complexes, which likely result from polymerization reactions of *ortho*-quinones formed. These insoluble complexes were not determined by UHPLC-UV-MS^n^. The complexation of *ortho*-quinones resulted, obviously, in a decrease of the UV signal of the substrates (Fig. 4; Additional file 5: Figure S4).

### Conversion of guaiacol and 3-methylcatechol by *Mt*PPO7

For a better discrimination between monophenolase and diphenolase activity, *Mt*PPO7 conversion of guaiacol and 3-methylcatechol was monitored over a period of 24 h by UHPLC-UV-MS^n^. The conversion of guaiacol by *Mt*PPO7 resulted in the initial formation of 3-methoxycatechol (Additional file 4: Table S1; Additional file 5: Figure S4, Additional file 6: Figure S5). Further reactions resulted in the formation of brown pigments indicating that 3-methoxycatechol was oxidized into *ortho*-quinones, which are likely to polymerize and form insoluble complexes. Other masses determined by UHPLC-UV-MS^n^ indicated the polymerization of guaiacol and compounds originating from the oxidation of guaiacol by *Mt*PPO7 into trimers (*m*/*z* 399, 401, 415). Based on the masses detected (Additional file 4: Table S1; Additional file 5: Figure S4), a scheme is presented of possible reaction pathways of guaiacol occurring during *Mt*PPO7 incubation (Additional file 6: Figure S5).

The oxidation of guaiacol by *Mt*PPO7 mainly occurred between 4 and 8 h of incubation, whereas 3-methylcatechol showed to be oxidized by *Mt*PPO7 within the first 2 h. During the incubation of 3-methylcatechol with *Mt*PPO7 pink pigments are formed, which precipitate after sample centrifugation. Based on MS^n^, masses of 3-methylcatechol (*m*/*z* 123) and masses indicating the dimerization of 3-methylcatechol (*m*/*z* 245) were detected upon incubation of both 3-methylcatechol with *Mt*PPO7 and 3-methylcatechol alone (Additional file 4: Table S1). No detectable amounts of new products were formed during the incubation of 3-methylcatechol with *Mt*PPO7 compared to the incubation of 3-methylcatechol only (Additional file 4: Table S1).

### Structural model of *Mt*PPO7

**Fig. 5:**
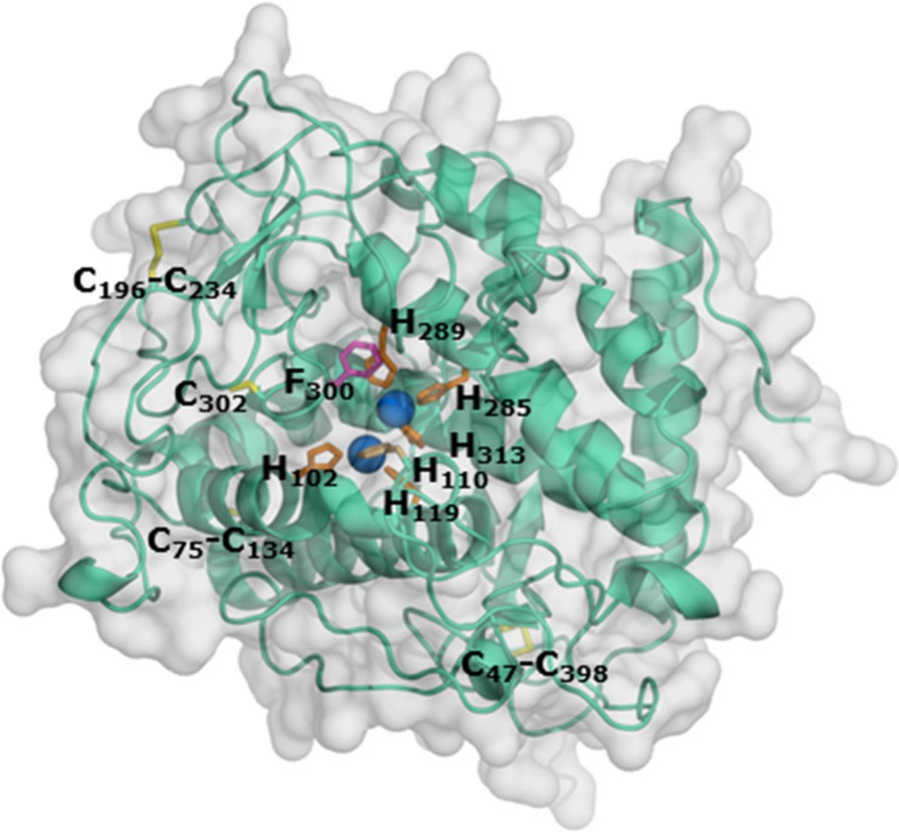
Structural model of *Mt*PPO7. The structural model of *Mt*PPO7 was generated based on the crystal structure of a catechol oxidase from *Aspergillus oryzae* (*Ao*CO4, Protein Data Bank entry: 4j3p) [42]. *Mt*PPO7 and *Ao*CO4 share an amino acid sequence identity of 38%. The *Mt*PPO7 model is predominantly α-helical with the catalytic copper site situated in the four-helix bundle. The coordination of the two copper atoms (*blue*) by six histidine residues (*orange*) is strictly conserved. The three disulfide bridges Cys47-Cys398, Cys75-Cys134, and Cys196-Cys234 (*yellow*) are also conserved

A structural model of *Mt*PPO7 was generated based on the available structure of a catechol oxidase from *Aspergillus oryzae* (*Ao*CO4, Protein Data Bank entry: 4j3p) (Fig. 5). *Mt*PPO7 and *Ao*CO4 share 38% amino acid sequence identity. The structural model of *Mt*PPO7 shows a four-helix bundle fold with the presence of six conserved histidines coordinating the two copper ions in the active site, which is typical for PPO-like tyrosinases and catechol oxidases. Six of the seven cysteines are involved in conserved disulfide bridges (Cys47–Cys393, Cys75–Cys134, and Cys196–Cys234) and expected to be relevant for the thermo-tolerance of *Mt*PPO7 (Fig. 5). Based on the model, the large distance (10.5 Å) between the sulfur atom of Cys302 and C*ɛ*-atom of His110 prevents formation of a thioether bond, which is present in other PPOs such as *Ab*PPO3 and *Ab*PPO4 [25]. Characteristics of the amino acid sequence and structural model of *Mt*PPO7 are further described in the “Discussion” section.

### Genome-wide analysis of AA9 LPMO, *Ab*PPO, and *Mt*PPO7

**Fig. 6:**
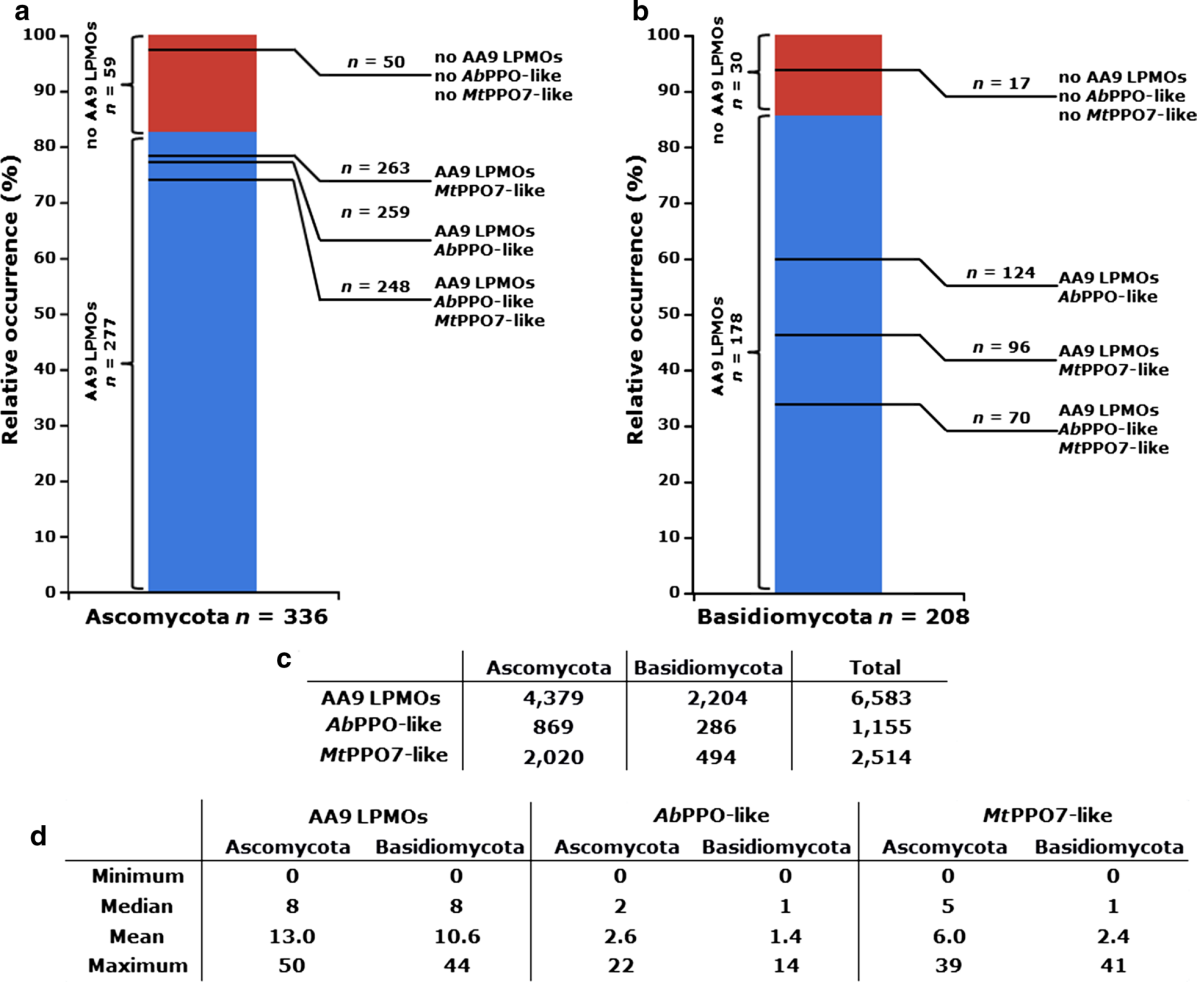
Genome analysis of Ascomycota and Basidiomycota. The protein sequence annotations of 336 Ascomycota and 208 Basidiomycota genomes were used for genome analysis. In total, **a** 277 Ascomycota and **b** 178 Basidiomycota comprise AA9 LPMOs encoding genes (*blue column*). Next to this occurrence of AA9 LPMOs encoding genes in Ascomycota and Basidiomycota, the co-occurrence of both *Ab*PPO-like and *Mt*PPO7-like, or either *Ab*PPO-like or *Mt*PPO7-like enzymes is indicated on the *right of each column*. The number of Ascomycota and Basidiomycota without co-occurrence of AA9 LPMOs encoding genes was 59 and 30, respectively (*red column*). In total, 50 Ascomycota and 17 Basidiomycota species were determined that do not carry any genes encoding AA9 LPMOs, *Ab*PPOs, and *Mt*PPO7s. **c** Total number of AA9 LPMOs, *Ab*PPOs, and *Mt*PPO7s encoding genes annotated in 336 Ascomycota and 208 Basidiomycota genomes. **d** Distribution of annotated AA9 LPMOs, *Ab*PPOs, and *Mt*PPO7s encoding genes within 336 Ascomycota and 208 Basidiomycota genomes. See “Methods” for details

Sequence annotations of 336 Ascomycota and 208 Basidiomycota genomes [29] were used in this analysis. Some of the numbers of genes obtained encoding AA9 LPMOs were verified by comparison with published data. For example, we have identified 22 and 18 genes encoding AA9 LPMOs in *M. thermophila* and *T. terrestris*, respectively, which is similar to previously reported results [30]. In total, 277 Ascomycota genomes and 178 Basidiomycota genomes contained AA9 LPMOs encoding genes (Fig. 6). The two PPOs used in this work are regarded as two different proteins due to their low sequence identity of 12%. The two PPO classes used for the genome analysis were not further divided into short and long tyrosinases [31]. Over 90% of the Ascomycota that comprise genes encoding AA9 LPMOs showed also the presence of genes encoding either *Ab*PPO-like, *Mt*PPO7-like proteins or both (Fig. 6a). In contrast, only around 40% of the AA9 LPMOs encoding Basidiomycota contained genes that encode *Ab*PPO-like, *Mt*PPO7-like proteins or both. The percentage of Ascomycota and Basidiomycota genomes studied that contained neither genes encoding AA9 LPMOs*, Ab*PPOs, nor *Mt*PPO7s were 15 and 8%, respectively (Fig. 6a). The average number of genes found per genome encoding AA9 LPMOs was higher in Ascomycota (13.0) than in Basidiomycota (10.6) (Fig. 6b, c). Both the total and the average number of genes encoding *Ab*PPO-like and *Mt*PPO7-like proteins were also higher in Ascomycota compared to Basidiomycota (Fig. 6b, c).

Principal component analysis (PCA) was performed on all 336 Ascomycota and 208 Basidiomycota. In addition, we used the numbers which describe the presence of AA9 LPMO, *Mt*PPO7s, and *Ab*PPOs encoding genes in each fungal species.

**Fig. 7:**
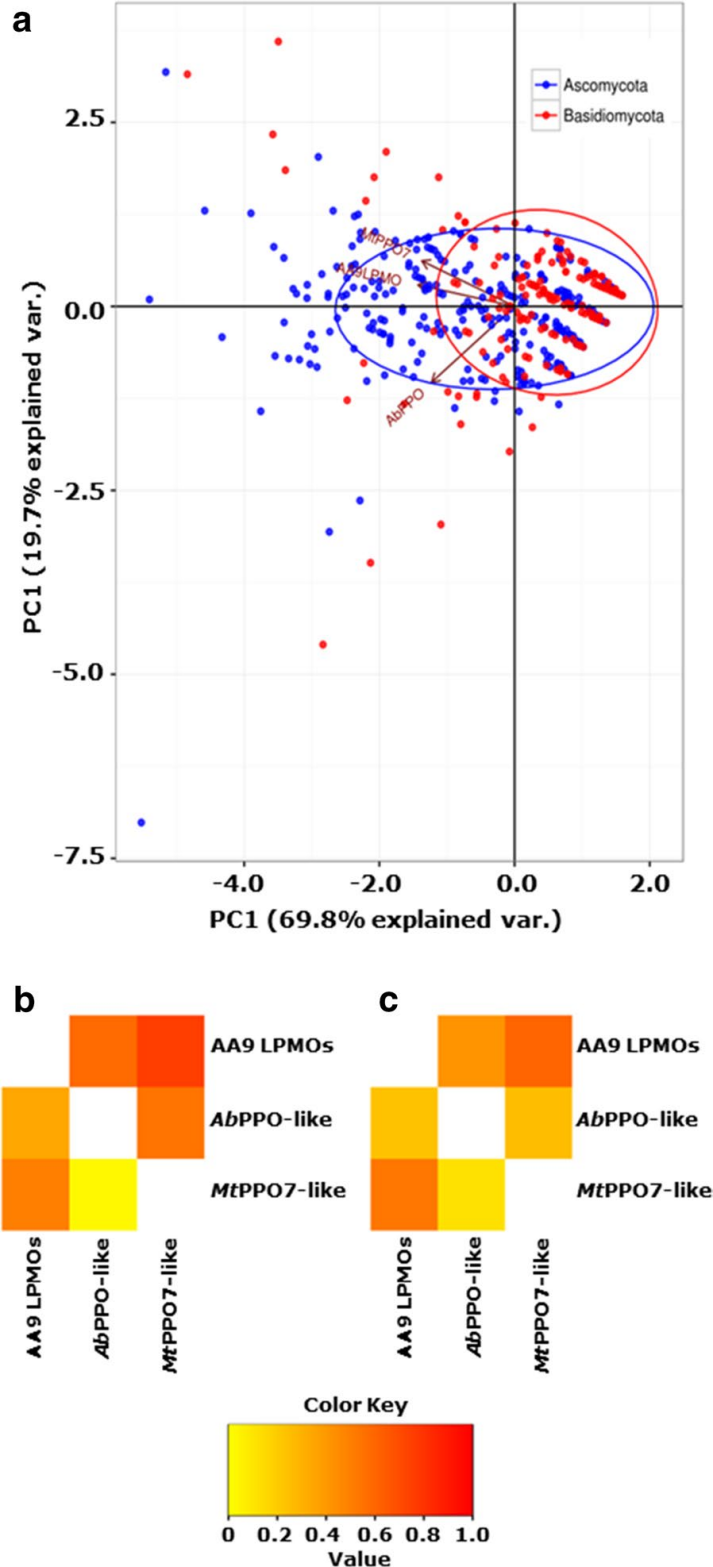
Statistical genome analysis of Ascomycota and Basidiomycota. **a** Principal component analysis (PCA) of genes encoding AA9 LPMOs, *Ab*PPOs, and *Mt*PPO7s of 336 Ascomycota and 208 Basidiomycota genomes. The numbers which describe the presence of genes encoding AA9 LPMOs, *Mt*PPO7s, and *Ab*PPOs in each fungal species were used as variables and are presented as principal components of Ascomycota (*red dots*) and Basidiomycota (*blue dots*). The first two components of the PCA explained 69.8 and 19.6% variation in the data, respectively. Vectors (*red arrows*) with a similar orientation illustrate a high correlation between the gene families (**b**). The axis legends indicate the overall contribution of the three gene families encoding AA9 LPMOs, *Ab*PPOs, and *Mt*PPO7s. **b** Correlation between the three gene families encoding AA9 LPMOs, *Ab*PPOs, and *Mt*PPO7s of 336 Ascomycota (*upper triangle*) and 208 Basidiomycota (*lower triangle*). **c** Correlation between genes encoding AA9 LPMOs, *Ab*PPOs, and *Mt*PPO7s of 27 Ascomycota (*upper triangle*) and 23 Basidiomycota (*lower triangle*), which have at least ten annotated genes encoding cellulose-degrading enzymes per fungus. Species of both fungal classes are listed in Additional file 7: Table S2 and Additional file 8: Table S3. Numerical values of the correlations of **b** and **c** are presented in Additional file 9: Figure S6. See “Methods” for details

The first two components of the PCA explained 69.8 and 19.7% variation in the data, respectively. Correlations of 0.75 and 0.5 were observed between the presence of genes encoding AA9 LPMOs and *Mt*PPO7s in Ascomycota and Basidiomycota, respectively (Fig. 7b). Lower correlations of 0.59 and 0.34 were observed in the genes encoding AA9 LPMOs and *Ab*PPOs. No correlation (correlation ≤0.25) was observed between *Ab*PPOs and *Mt*PPO7, for both Ascomycota and Basidiomycota. Based on the presence of at least 10 annotated genes that encode cellulose-degrading enzymes per fungus, 27 Ascomycota and 23 Basidiomycota genomes were selected (Additional file 7: Table S2, Additional file 8: Table S3) [32]. The selected 27 Ascomycota genomes showed a higher correlation (0.60) between genes encoding AA9 LPMOs and *Mt*PPO7s compared to the 23 selected Basidiomycota (0.53) (Fig. 7c). Also, no correlation was found between genes encoding *Mt*PPO7- and *Ab*PPO-like genes in the selected Basidiomycota species (Fig. 7).

## Discussion

### *Mt*PPO7 enhances cellulose oxidation by *Mt*LPMO9B

Many PPOs, tyrosinases in particular, have been described to oxidize monophenols and various phenolic compounds comprising a 1,2-benzenediol or 1,2,3-benzenetriol moiety. However, none of these tyrosinases show a high activity towards methoxylated compounds (group Ib) such as ferulic acid (no. 8) [33–36]. Importantly, *Mt*PPO7 hydroxylates methoxylated phenolic compounds (group Ib) and thereby improves the activity of *Mt*LPMO9B up to 75 times (no. 5, Table 1). On the other hand, *Ab*PPO addition to RAC incubated with *Mt*LPMO9B in the presence *non*-*methoxylated* monophenols also resulted in a significant increase of the *Mt*LPMO9B activity (up to 99 times). Especially the activity of *Mt*PPO7 towards methoxylated phenolic compounds is of high relevance, as these compounds are abundant as structural lignin units and, therefore, intrinsically present during plant biomass degradation. Here, we only determined the effect of polyphenol oxidases on the LPMO-mediated cellulose degradation in the presence of phenolic compounds that are known as lignin building blocks but we did not, for example, investigate the impact of these enzymes in the presence of washed or unwashed pretreated biomass. In comparison to the substrate used in this thesis, we expect a lower effect of *Mt*PPO7-like enzymes on the LPMO-mediated substrate oxidation using pretreated biomass, since this biomass contains a mixture of multiple potential electron-donating compounds, which were described in an earlier study [13].

A recent study described that H_2_O_2_, in addition to or instead of O_2_ (Fig. 1), acts as a co-substrate for LPMOs, while reducing agents are still needed to activate the active site copper [37]. Once copper is activated, LPMOs oxidize substrates under the use of H_2_O_2_. The latter can be formed by the reduction of O_2_ through reducing agents [37]. Considering these recent findings, it can only be hypothesized how *Mt*PPO7 increased the *Mt*LPMO9B-mediated cellulose oxidation, which was investigated in our research. It is possible that the oxidation of the phenolic compounds led to the formation of compounds that have either an enhanced reducing efficiency on *Mt*LPMO9B or led to an enhanced H_2_O_2_ generation which increased the *Mt*LPMO9B activity.

### Activity of *Mt*PPO7 towards phenolic compounds

Based on our results, we conclude that *Mt*PPO7 improves the activity of *Mt*LPMO9B by two main reactions. First, *Mt*PPO7 hydroxylates methoxylated monophenols at the *ortho*-position (monophenolase activity) and forms compounds comprising a 1,2-benzenediol moiety. These compounds have, compared to methoxylated monophenols, a lower redox potential and are known to be good electron donors for LPMOs [12–14]. Secondly, *Mt*PPO7 inhibits the formation of non-enzymatic coupling reactions, which occurred during the incubation of several cinnamic acid derivatives in the absence of *Mt*PPO7, but were not formed when *Mt*PPO7 was present during the incubations (Additional file 4: Table S1). Non-enzymatic coupling reactions of cinnamic acid derivatives of group I, II, or III result in the formation of bulky phenolic polymers. The aliphatic acrylic acid (prop-2-enoic acid) group present in cinnamic acid derivatives constitutes an elongation of the conjugated aromatic ring. This group takes part in polymerization reactions, which can be caused by radical formation due to the presence of copper (2.5 µM) [18, 38]. However, it remains unclear, how *Mt*PPO7 prevents (*reduces*) the polymerization of cinnamic acid derivatives.

**Fig. 8:**
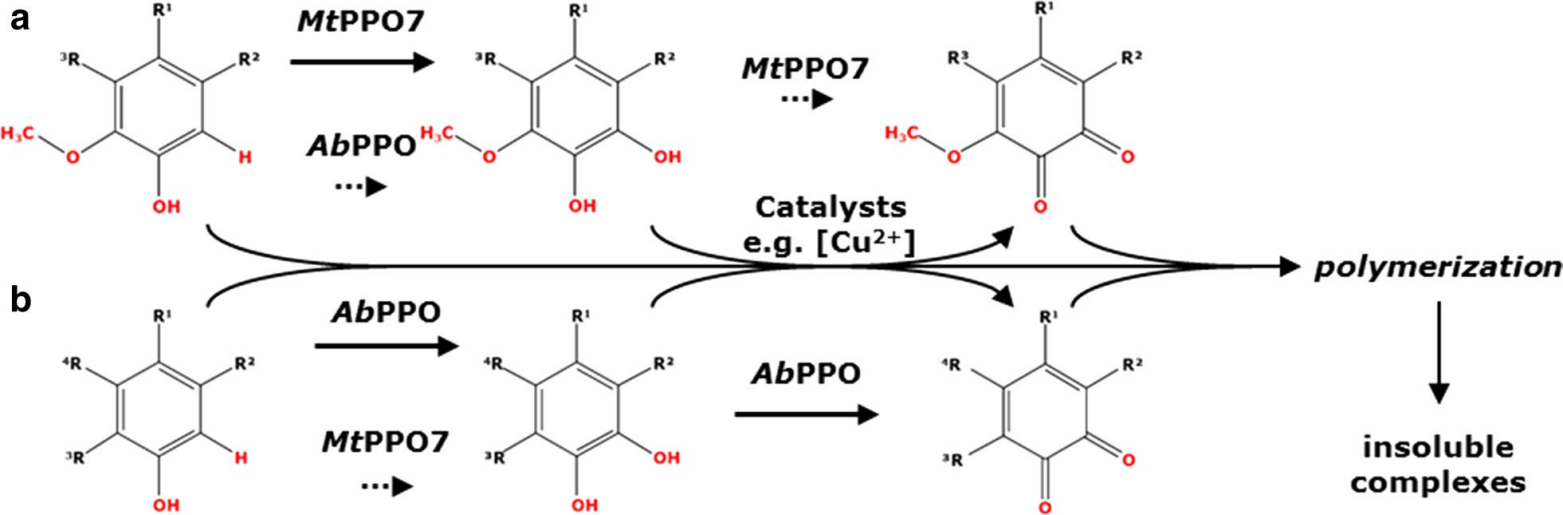
Proposed reaction pathway of *Mt*PPO7- and *Ab*PPO-mediated oxidation of phenolic compounds. **a** Phenolic compounds comprising a 2-methoxy moiety are hydroxylated by *Mt*PPO7 from *M. thermophila* C1 at the 6-position. Different from *Mt*PPO7, *Ab*PPO from *A. bisporus* shows a low efficiency (*dotted arrows*) towards these methoxylated monophenols. The formed compounds comprising a 1,2-dihydroxy-3-methoxy moiety have, compared to the methoxylated monophenols, a lower electron potential and an increased electron-donating capacity for LPMOs. **b** Non-methoxylated monophenols are hydroxylated at the *ortho*-position by *Ab*PPO into compounds comprising a 1,2-benzenediol moiety, whereas *Mt*PPO7 shows a low efficiency (*dotted arrows*) towards non-methoxylated monophenols. The formed compounds comprising a **a** 1,2-dihydroxy-3-methoxy and **b** 1,2-benzenediol moiety are expected to be further oxidized into *ortho*-quinones by either the *Mt*PPO7- and *Ab*PPO-mediated or catalyst-mediated oxidation, such as copper which was present during the incubation. As indicated in Additional file 6: Figure S5, these *ortho*-quinones, including intermediate products formed by the oxidation of phenolic compounds, are expected to polymerize and form insoluble complexes

Importantly, *Mt*PPO7 showed only a low efficiency towards non-methoxylated compounds comprising a 1,2-benzenediol or 1,2,3-benzenetriol moiety. In contrast to *Ab*PPO, *Mt*PPO7 had no inhibitory effect on the *Mt*LPMO9B activity towards RAC in the presence of these phenolic compounds (Table 1). Although we did not determine the formation of *ortho*-quinones, it is possible that *Mt*PPO7 further oxidizes methoxylated compounds comprising a 1,2-benzenediol moiety into *ortho*-quinones due to the observed pigment formation (Additional file 5: Figure S4, Additional file 6: Figure S5). However, this pigment formation could also partly result from non-enzymatic polymerization reactions or formation of metal-catechol complexes, as indicated in Fig. 8 [39]. Still, based on the monophenolase activity we would consider *Mt*PPO7 to be a tyrosinase. The low efficiency of *Mt*PPO7 to convert compounds comprising a 1,2-benzenediol moiety is expected to be an advantage. *Mt*LPMO9B can utilize these compounds as electron donors and improve the oxidative activity towards cellulose. In the case of a strong diphenolase activity, however, the available amount of these compounds comprising a 1,2-benzenediol moiety would rapidly decrease due to further oxidation of these compounds into *ortho*-quinones. Hence, we expect for some other PPOs, such as *Ab*PPO, a negative effect on the LPMO-mediated cellulose oxidation. This effect results from the strong activity of these PPOs towards compounds comprising a 1,2-benzenediol or 1,2,3-benzenetriol moiety, which are potential reducing agents for LPMOs [33, 40, 41]. The *ortho*-quinones formed (no. 17 and 18) (Fig. 2; Table 1) have been shown to be less efficient electron donors for LPMOs than their *ortho*-diphenol precursors [12].

### Structural characteristics of *Mt*PPO7

The structural model of *Mt*PPO7 shows a four-helix bundle architecture with the presence of six histidine residues coordinating the two copper ions in the active site, which is typical for PPO-like tyrosinases and catechol oxidases (Fig. 5). We clearly showed that the preference of *Mt*PPO7 towards methoxylated phenolic compounds and the low activity towards diphenols is different from reported fungal or plant catechol oxidases and tyrosinases (Fig. 4). Here, we further discuss the structure–function relationship of these PPOs in comparison with the generated model of *Mt*PPO7. However, it should be noted that the structural model of *Mt*PPO7 was generated based on a catechol oxidase from *Aspergillus oryzae* (*Ao*CO4) and both proteins share an overall amino acid sequence identity of only 38% [42]. It has already been shown that marginal structural differences determine whether PPOs exhibit a stronger monophenolase or diphenolase activity [43]. Based on our model, it is possible that structural features discussed below deviate compared to the crystal structure which is, however, not available yet.

Firstly, the presence of a thioether bridge between a cysteine and histidine is assumed to be important for the monophenolase and diphenolase activity. This bond has been described to be present in many fungal PPOs, such as *Ab*PPO3/4 from *A. bisporus*, *Nc*PPO from *Neurospora crassa,* and also in plant catechol oxidases, such as *Ib*CO from *Ipomoea batatas* [26, 44, 45]. However, similar to *Ao*CO4 from *A. oryzae* [42], *Mt*PPO7 does not form this thioether bond (Fig. 5). Secondly, *Mt*PPO7 shows the presence of the PPO typical ‘gate residue’ (Phe300 in *Mt*PPO7, Phe261 in *Ib*CO, and Val299 in *Ao*CO4). It has been hypothesized that a bulky ‘gate residue,’ such as a phenylalanine, limits the accessibility of monophenols to the copper A ion (CuA) and, therefore, might be a limiting factor for the monophenolase activity [44, 46]. However, in *Ao*CO4 the bulky phenylalanine is replaced by Val299 and still *Ao*CO4 shows a rather low activity towards monophenols [31, 42]. Our data suggest that Phe300 in *Mt*PPO7 does not limit the accessibility for (methoxylated) monophenols. Thirdly, it also has recently been proposed that the presence of certain amino acid residues next to the CuB-coordinating histidines indicates whether a tyrosinase or catechol oxidase exhibits a stronger monophenolase and a weak diphenolase activity or a weak monophenolase and a strong diphenolase activity [43]. However, none of the described amino acid residues at that specific position (Pro, Asn, Glu, and Gln) is present in *Mt*PPO7, which has a tyrosine (Tyr268) at the corresponding position. In *Mt*PPO7 a tyrosine (Tyr268) occupies. Whether Tyr268 is responsible for the observed specificity towards methoxylated monophenols can only be hypothesized at this moment. Fourthly, *Mt*PPO7 shares with tyrosinases and catechol oxidases the characteristic ‘tyrosine motif’ (Tyr-X-Tyr/Phe or Tyr/Phe-X-Tyr; in *Mt*PPO7 residues 399-401). This motif is highly conserved among plants and fungi [31, 42, 47]. Fifthly, *Mt*PPO7 does not contain the ‘YG motif’ (Gly-Tyr motif), which is a typical feature of fungal tyrosinases and catechol oxidases [47]. In conclusion, based on substrate specificity (Fig. 1) and modeled structure (Fig. 5), we do not classify *Mt*PPO7 as a typical tyrosinase or catechol oxidase.

### Genome analysis

Based on genome analysis, we found a positive correlation between genes encoding AA9 LPMOs and *Mt*PPO7-like enzymes in Ascomycota and Basidiomycota, which has not been shown previously. Interestingly, this correlation strengthens the evidence that fungi benefit from the concerted activity of AA9 LPMOs and *Mt*PPO7-like enzymes in nature, which is in agreement with major findings obtained from the experimental data of this work. The proportion of annotated AA9 LPMOs within Ascomycota and Basidiomycota (82 and 86%, respectively) is about 5–10% lower with the findings of a recent study (92% in Ascomycota and Basidiomycota), which may result from the different species chosen (Fig. 6) [12]. The two PPOs (*Mt*PPO7 and *Ab*PPO) used in this work share only a low sequence identity and, therefore, are considered to belong to different subgroups of polyphenol oxidases. It is important to realize that active tyrosinases, such as the mushroom tyrosinase *Ab*PPO3, have been described to consist of large (H) and small (L) subunits [45, 48]. We focused for the genome analysis on genes encoding the protein of the catalytic H subunit, which contains the highly conserved binuclear copper site. The function of the L subunit is so far not known [45]. All purified enzymes used in this work are secreted. However, we did not differentiate between secreted and non-secreted fungal AA9 LPMOs and PPOs in the genome analysis. It may be that most of the enzymes detected here take part in the external fungal metabolism, as can be deducted from the high abundance of the annotated *Mt*PPO7s and *Ab*PPOs throughout Ascomycota and Basidiomycota. We, therefore, specifically selected genomes of plant biomass degrading fungi that contain at least ten genes encoding known cellulose-degrading enzymes per fungus and found again a similar correlation between genes encoding AA9 LPMOs, *Ab*PPOs, and *Mt*PPO7s (Fig. 7) [32, 49]. In contrast to *Mt*PPO7s, genes encoding *Ab*PPOs showed a weak correlation with AA9 LPMOs in Ascomycota and Basidiomycota (Fig. 7), especially for the selected cellulose-degrading Basidiomycota (Fig. 7c). One possible explanation is that a cellulose-rich environment is associated with the abundance of lignin as seen in soft- and hardwoods. Still, there may be considerable variation in the substrate specificity and specific activity of PPOs within the *Mt*PPO7- or *Ab*PPO-like gene families. The latter seems to be consistent with the low sequence identity among PPOs and, in addition, by the diverse substrate specificities of PPOs that have only marginal differences in their amino acid sequence [31, 43, 50].

## Conclusions

For the first time, we demonstrate the importance of the coupled action of different monooxygenases in the concerted degradation of plant biomass. We demonstrated that *Mt*PPO7 is particularly active towards methoxylated phenolic compounds that are the predominant structural units of lignin. This feature distinguishes *Mt*PPO7 from the well-known mushroom tyrosinase *Ab*PPO, which stimulates the LPMO activity via its ability to hydroxylate non-methoxylated monophenols. However, its strong diphenolase activity limits the applicability of this tyrosinase for producing electron-donating capacity for LPMOs. In addition, we established that genes encoding *Mt*PPO7-like enzymes and AA9 LPMOs are highly correlated throughout Ascomycota and Basidiomycota, suggesting that AA9 LPMOs benefit from the activity of *Mt*PPO7-like enzymes in nature. Further understanding in both lignin deconstruction and enzymatic lignocellulose oxidation will lead to more eco-friendly routes for biomass utilization, which is seen as a prerequisite for a circular economy.

## Methods

### Enzyme expression, production, and purification

*Mt*PPO7 (UniProt: KX772412) was over-expressed in the homologous *Myceliophthora thermophila* C1 strain. A low protease/low (hemi-)cellulase-producing *M. thermophila* C1 strain was used to produce *Mt*PPO7 [51, 52]. The *Mt*PPO7-containing culture broth was fractionated to obtain a pure *Mt*PPO7 preparation. This preparation was provided by DuPont Industrial Biosciences. *Mt*LPMO9B was expressed and purified as described in [13].

### Protein identification

The pure *Mt*PPO7 fraction was analyzed by LC–mass spectrometry confirming the presence of the *Mt*PPO7 by ‘The Scripps Research Institute’ (San Diego, CA, USA).

### Purification and identification of mushroom tyrosinase

Tyrosinase from the edible button mushroom *Agaricus bisporus* was purified from a commercial enzyme preparation (Sigma-Aldrich, Steinheim, Germany) as described previously [25]. The purified enzyme preparation (referred to as *Ab*PPO) was shown to contain the isoforms PPO3 and PPO4 [25].

### Cellulose substrate and reducing agents

Regenerated amorphous cellulose (RAC) was prepared from Avicel PH-101 as described previously [7, 53]. All reducing agents used throughout this study (Table 1) were purchased from Sigma-Aldrich (Steinheim, Germany).

### Incubation conditions for *Mt*LPMO9B, *Mt*PPO7, and *Ab*PPO

Regenerated amorphous cellulose (1–2 mg mL^−1^, see Figure captions) was dissolved in 50 mM ammonium acetate buffer (pH = 5.0), with or without addition of reducing agents (final concentration of 2 mM). The standard enzyme concentrations of *Mt*LPMO9B, *Mt*PPO7, and *Ab*PPO used in this work were 5.0, 5.0, and 0.7 µg protein mg^−1^ substrate, respectively. All samples were incubated for 20 h at 50 °C in a head-over-tail rotator in portions of 1 mL total volume (Stuart rotator, Bibby Scientific, Stone, UK) at 20 rpm. Supernatants of all incubations were analyzed by HPAEC and RP-UHPLC-UV-ESI–MS^n^.

### Oligosaccharide analysis

Oligosaccharides were analyzed by high-performance anion-exchange chromatography (HPAEC) with pulsed amperometric detection (PAD) using a HPAEC system (ICS-5000, Dionex, Sunnyvale, CA, USA) as described previously [7].

### RP-UHPLC-UV-ESI-MS^n^ analysis

Supernatants of all incubations were subjected to an Accela reversed-phase high-performance liquid chromatography (RP-UHPLC) system coupled to electron spray ionization mass spectrometry (Thermo Scientific, San Jose, CA, USA). Injected samples (5 μL) were separated using an Acquity C18 column (2.1 × 150 mm, 1.7 μm particle size) with an Acquity UHPLC Shield RP18 Vanguard guard column (2.1 × 5 mm, 1.7 μm particle size). Both columns were purchased from Waters (Milford, MA, USA). Gradient elution with eluent A (H_2_O + 1% (v/v) acetonitrile + 0.1% (v/v) HOAc) and eluent B (acetonitrile + 0.1% (v/v) HOAc) was performed according to the following steps: From 0 to 17.7 min a linear gradient from 5 to 60% B; from 17.7 to 21.7 min, isocratic 100% B, and from 21.7 to 26 min, isocratic 5% B. The flow rate and the injection volume were 0.4 mL min^−1^ and 5 μL, respectively. The column temperature was set to 30 °C and the photodiode array detector was operated in the range of 200–400 nm.

Samples were further analyzed using an LTQ-Velos mass spectrometer (Thermo Scientific) equipped with a ESI–MS. Data were collected over a *m/z* range of 90–1500 in both negative (NI) and positive (PI) modes. The collision energy was set to 35%.

### Structural modeling

An alignment was made of the amino acid sequence of *Mt*PPO7 and the amino acid sequence of catechol oxidase from *Aspergillus oryzae* (*Ao*CO4), which scored highest in a Blast search using the *Mt*PPO7 sequence against the Protein Data Bank (38% amino acid identity). Using this alignment and the available structure of *Ao*CO4 (PDB id: 4J3P) as template, structural models were obtained for *Mt*PPO7 using the Modeller program version 9.16. One hundred comparative models were generated, after which the model with lowest corresponding DOPE score was selected for inspection and image generation using Pymol (Pymol, The PyMOL Molecular Graphics System, version 1.5.0.4 Schrödinger, LLC, New York, NY, USA).

### Genome-wide analysis

Fungal genomes were obtained from the JGI MycoCosm portal [29]. In total, protein sequence annotations of 336 Ascomycota and 208 Basidiomycota genomes were used. BLAST databases for those protein sequences were created using ‘makeblastdb’ program in BLAST + v2.2.30 [54]. The protein BLAST was performed separately using ten *Mt*PPO7 sequences [PDB id: 4j3p and closely related *Mt*PPO7s: Q2UNF9, *Aspergillus oryzae* (strain ATCC 42149); G2QC95, *Myceliophthora thermophila* (ATCC 42464); Q2H7I7, *Chaetomium globosum* (ATCC 6205); G0SFX8, *Chaetomium thermophilum* (DSM 1495); L7IAQ4, *Magnaporthe oryzae* (strain Y34); L7JMT9, *Magnaporthe oryzae* (strain P131); G4N2I5, *Magnaporthe oryzae* (strain 70-15); A0A084GCK1, *Scedosporium apiospermum*; A0A0C4DYF2, *Magnaporthiopsis poae* (ATCC 64411); J3P591, *Gaeumannomyces graminis var. tritici* (strain R3-111a-1)], ten AA9 LPMO sequences (PDB id: 4d7u and closely related LPMOs: Q7SHI8, *Neurospora crassa* (strain ATCC 24698); G2QCJ3, *Myceliophthora thermophila* (strain ATCC 42464); F7W1P4, *Sordaria macrospora* (strain ATCC MYA-333); G2RB73, *Thielavia terrestris* (strain ATCC 38088); Q2H8N9, *Chaetomium globosum* (strain ATCC 6205); G0S408, *Chaetomium thermophilum* (strain DSM 1495); F8MLY8, N*eurospora tetrasperma* (strain FGSC 2508); T0L448, *Colletotrichum gloeosporioides* (strain Cg-14); A0A0H4K9X4 and A0A1C9CXI0, *Myceliophthora thermophila* C1); and four *Ab*PPOs (*Ab*PPO1, *Ab*PPO2, *Ab*PPO3, and *Ab*PPO4 [55, 56]) as query sequences. Resulting sequences below E-value cut-off of 0.001 with query coverage above 60% for AA9 LPMOs, 65% for *Mt*PPO7s, and 85% for *Ab*PPOs were considered for further analysis. Selection of cellulase-rich Ascomycota and Basidiomycota was based on the presence of at least 10 genes encoding cellulose-degrading enzymes, which are classified in the CAZy database as glycosyl hydrolase families GH1, GH3, GH5, GH6, GH7, GH12, GH45. The GH gene families were selected based on Kubicek et al. [57]. Previous data [32] were used to determine the number of annotated genes encoding cellulose-degrading enzymes. Based on this selection, 27 Ascomycota and 23 Basidiomycota species were selected for the Pearson correlation analysis (Fig. 7; Additional file 7: Table S2, Additional file 8: Table S3). All the statistical analyses were performed in R [58].

## Additional files

**Additional file 1: Figure S1.** Activity of *Mt*LPMO9B towards amorphous cellulose in the presence and absence of *Mt*PPO7 or *Ab*PPO. HPAEC elution pattern of regenerated amorphous cellulose (RAC; 1.5 mg mL^−1^) incubated with *Mt*LPMO9B (red, 5.0 μg mL^−1^) only, or with either *Ab*PPO (blue, 2.5 µL mL^−1^) or *Mt*PPO7 (yellow, 5.0 μg mL^−1^) in the presence of (**a**) *para*-coumaric acid (no. 3 specified in Table 1, 2 mM) and (**b**) 3-hydroxy-4-methoxycinnamic acid (no. 5 specified in Table 1, 2 mM). The incubation of RAC with *Mt*LPMO9B results in the formation of non-oxidized gluco-oligosaccharides (GlcOS_n_) and C1-oxidized gluco-oligosaccharides (GlcOS_n_^#^). See “Methods” for details.

**Additional file 2: Figure S2.** Release of oligosaccharides from RAC incubated with *Mt*LPMO9B in the presence and absence of *Mt*PPO7 throughout 24 h. Samples were incubated in the presence of ferulic acid (no. 8 specified in Table 1). The total sum is shown as integrated peak areas of released non-oxidized (shaded red and shaded yellow) and C1-oxidized (red and yellow) gluco-oligosaccharides after incubation of regenerated amorphous cellulose (RAC; 1.5 mg mL^−1^) with *Mt*LPMO9B only (red bars, 5 mg mL^−1^) and *Mt*LPMO9B together with *Mt*PPO7 (yellow bars, 5 mg mL^−1^) based on HPAEC. All incubations were performed in duplicate, and the standard deviations are presented as error bars. See “Methods” for details.

**Additional file 3: Figure S3.** Concentration of phenolic compounds incubated in the presence and absence of *Mt*PPO7. (**a**) guaiacol (no. 9 specified in Table 1, 2 mM) and (**c**) 3-methylcatechol (no. 17 specified in Table 1, 2 mM) were incubated with *Mt*PPO7 (yellow bar, 5 μg mL^−1^) or without (red bar). Samples were incubated in a 50 mM potassium phosphate buffer (pH = 6.0) containing 2.5 µM copper(II)-chloride for 24 h at 50 °C. The conversion of guaiacol and 3-methylcatechol by *Mt*PPO7 was calculated by subtracting the determined concentration of the incubation of guaiacol or 3-methylcatechol in the presence of *Mt*PPO7 from the concentration that was determined by the incubation of guaiacol and 3-methylcatechol alone. This conversion was expressed as the relative decrease of the guaiacol and 3-methylcatechol concentration and is shown in (**b**) and (**d**), respectively. See “Methods” for details.

**Additional file 4: Table S1.** Compounds detected after incubation of 21 reducing agents without or with *Mt*PPO7 by UHPLC/UV-MS^1^.

**Additional file 5: Figure S4.** UHPLC-UV-MS^n^ elution profile of guaiacol incubated (a) in the presence and (b) absence of *Mt*PPO7. Guaiacol (no. 9 specified in Table 1, 2 mM) was incubated with (5 μg mL^−1^) or without *Mt*PPO7. Samples were incubated in a 50 mM potassium phosphate buffer (pH = 6.0) containing 2.5 µM copper(II)-chloride for 24 h at 50 °C. Annotation of the peaks based on UV was done by using mass spectrometry (Additional file 6: Figure S5). See “Methods” for details.

**Additional file 6: Figure S5.** Schematic presentation of possible reaction pathways of guaiacol incubated in the presence of *Mt*PPO7. In short, *Mt*PPO7 hydroxylates guaiacol (no. 9 specified in Table 1) into 3-methoxycatechol (monophenolase activity). Although not determined, it is likely that 3-methoxycatechol is further oxidized by *Mt*PPO7 into the corresponding *ortho*-quinone (diphenolase activity, dashed arrow). These *ortho*-quinones are expected to polymerize and form insoluble complexes. Guaiacol itself forms insoluble complexes via auto-oxidation, which results from the presence of copper during the incubation for 24 h at 50 °C. The decrease in guaiacol concentration during the incubation of guaiacol without *Mt*PPO7 is also shown in Additional file 3: Figure S3a. The determined masses indicate the presence of multiple trimers (399.06, 401.06 and 415.04) consisting of polymerized 3-methylcatechol and guaiacol (Additional file 5: Figure S4). As described above, the polymerization reactions are expected to be catalyzed by copper during the incubation conditions applied. All masses were determined by UHPLC-UV-MS^n^ after incubation of guaiacol (2 mM) with *Mt*PPO7 (5.0 μg mL^−1^). Samples were incubated for 24 h at 50 °C in 50 mM potassium phosphate, pH 6.0, containing 2.5 µM copper(II)-chloride.

**Additional file 7: Table S2.** Selected cellulase-rich Ascomycota from the JGI database^1^.

**Additional file 8: Table S3.** Selected cellulase-rich Basidiomycota from the JGI database^1^.

**Additional file 9: Figure S6.** Correlation of AA9 LPMOs, *Ab*PPOs and *Mt*PPO7s encoding genes in Ascomycota and Basidiomycota. Correlation between the three gene families encoding AA9 LPMOs, *Ab*PPOs and *Mt*PPO7s of (**a**) 336 Ascomycota and (**b**) 208 Basidiomycota. Correlation between genes encoding AA9 LPMOs, *Ab*PPOs and *Mt*PPO7s of (**c**) 27 Ascomycota and (**d**) 23 Basidiomycota, which have at least ten annotated genes encoding cellulose-degrading enzymes. Species of selected fungal classes are listed in Additional file 7: Table S2 and Additional file 8: Table S3. Graphical presentations of the correlations of (a) together with (b) and (c) and (d) are shown in Fig. 7. See “Methods” for details.
