# Supplementary figures and images for: Boosting LPMO-driven lignocellulose degradation by polyphenol oxidase-activated lignin building blocks
Source: Biotechnol Biofuels. 2017 May 10;10:121. doi: 10.1186/s13068-017-0810-4 (PMC5424327; doi:10.1186/s13068-017-0810-4)

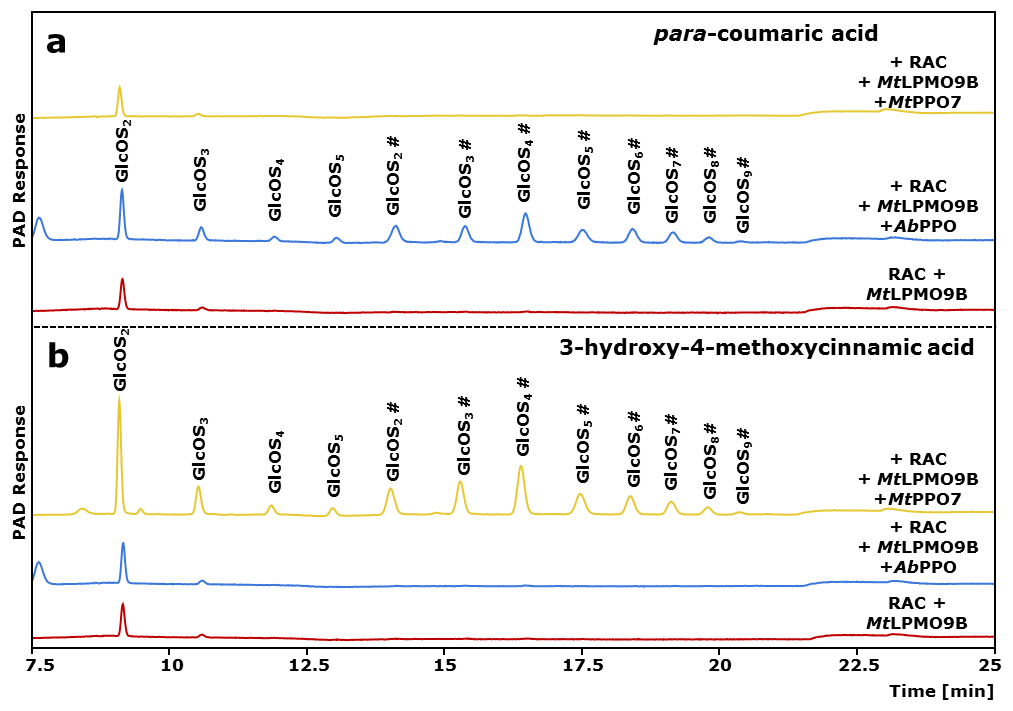

Supplement: Supplementary file 1 — Additional file 1: Figure S1. Activity of MtLPMO9B towards amorphous cellulose in the presence and absence of MtPPO7 or AbPPO. HPAEC elution pattern of regenerated amorphous cellulose (RAC; 1.5 mg mL−1) incubated with MtLPMO9B (red, 5.0 μg mL−1) only, or with either AbPPO (blue, 2.5 µL mL−1) or MtPPO7 (yellow, 5.0 μg mL−1) in the presence of (a) para-coumaric acid (no. 3 specified in Table 1, 2 mM) and (b) 3-hydroxy-4-methoxycinnamic acid (no. 5 specified in Table 1, 2 mM). The incubation of RAC with MtLPMO9B results in the formation of non-oxidized gluco-oligosaccharides (GlcOSn) and C1-oxidized gluco-oligosaccharides (GlcOSn#). See “Methods” for details. [file 13068_2017_810_MOESM1_ESM.png]

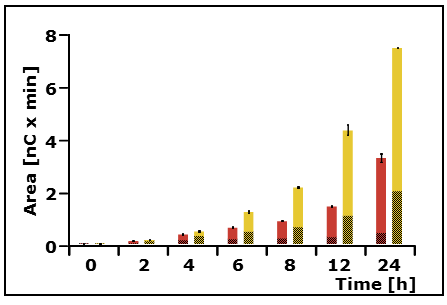

Supplement: Supplementary file 2 — Additional file 2: Figure S2. Release of oligosaccharides from RAC incubated with MtLPMO9B in the presence and absence of MtPPO7 throughout 24 h. Samples were incubated in the presence of ferulic acid (no. 8 specified in Table 1). The total sum is shown as integrated peak areas of released non-oxidized (shaded red and shaded yellow) and C1-oxidized (red and yellow) gluco-oligosaccharides after incubation of regenerated amorphous cellulose (RAC; 1.5 mg mL−1) with MtLPMO9B only (red bars, 5 mg mL−1) and MtLPMO9B together with MtPPO7 (yellow bars, 5 mg mL−1) based on HPAEC. All incubations were performed in duplicate, and the standard deviations are presented as error bars. See “Methods” for details. [file 13068_2017_810_MOESM2_ESM.png]

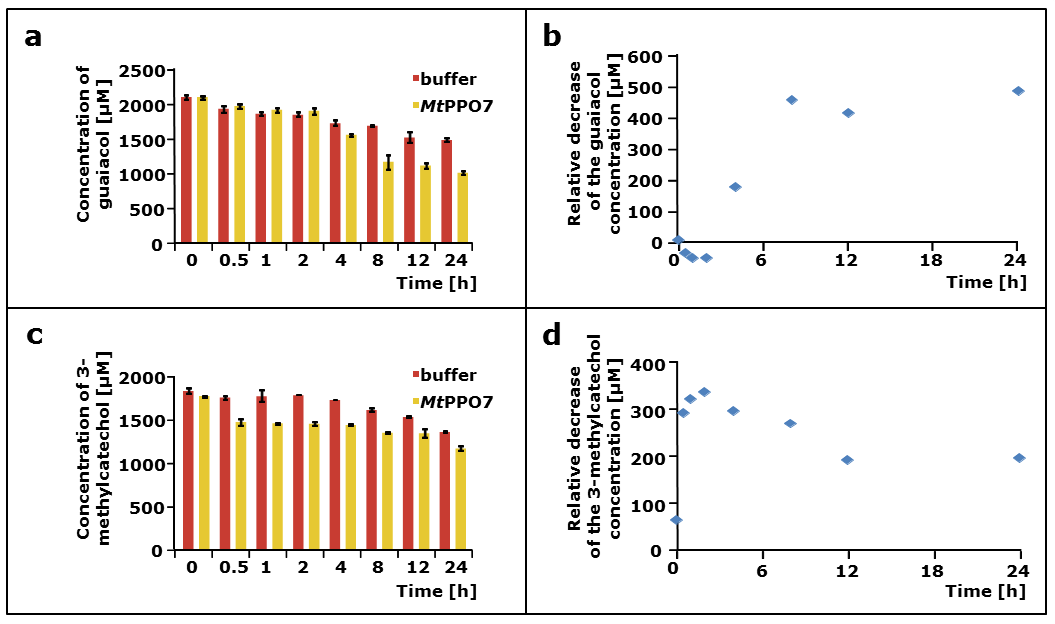

Supplement: Supplementary file 3 — Additional file 3: Figure S3. Concentration of phenolic compounds incubated in the presence and absence of MtPPO7. (a) guaiacol (no. 9 specified in Table 1, 2 mM) and (c) 3-methylcatechol (no. 17 specified in Table 1, 2 mM) were incubated with MtPPO7 (yellow bar, 5 μg mL−1) or without (red bar). Samples were incubated in a 50 mM potassium phosphate buffer (pH = 6.0) containing 2.5 µM copper(II)-chloride for 24 h at 50 °C. The conversion of guaiacol and 3-methylcatechol by MtPPO7 was calculated by subtracting the determined concentration of the incubation of guaiacol or 3-methylcatechol in the presence of MtPPO7 from the concentration that was determined by the incubation of guaiacol and 3-methylcatechol alone. This conversion was expressed as the relative decrease of the guaiacol and 3-methylcatechol concentration and is shown in (b) and (d), respectively. See “Methods” for details. [file 13068_2017_810_MOESM3_ESM.png]

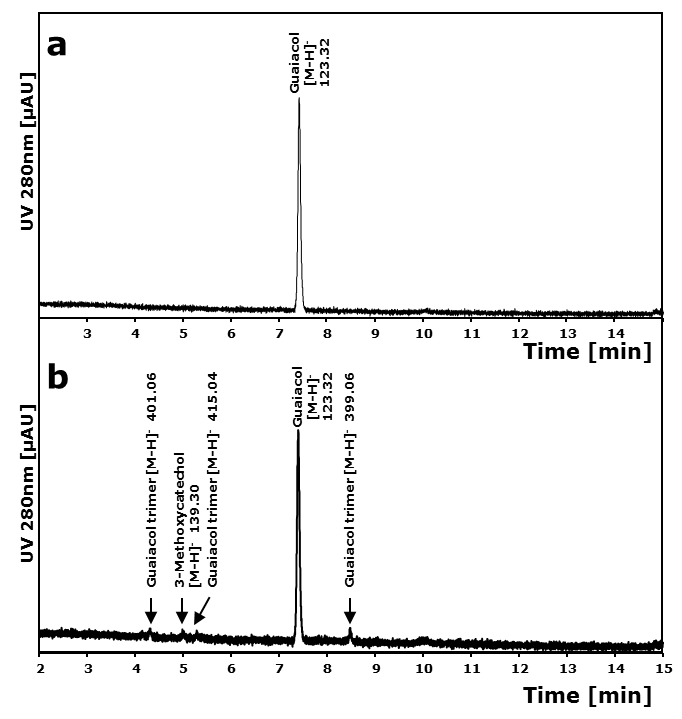

Supplement: Supplementary file 5 — Additional file 5: Figure S4. UHPLC-UV-MSn elution profile of guaiacol incubated (a) in the presence and (b) absence of MtPPO7. Guaiacol (no. 9 specified in Table 1, 2 mM) was incubated with (5 μg mL−1) or without MtPPO7. Samples were incubated in a 50 mM potassium phosphate buffer (pH = 6.0) containing 2.5 µM copper(II)-chloride for 24 h at 50 °C. Annotation of the peaks based on UV was done by using mass spectrometry (Additional file 6: Figure S5). See “Methods” for details. [file 13068_2017_810_MOESM5_ESM.png]

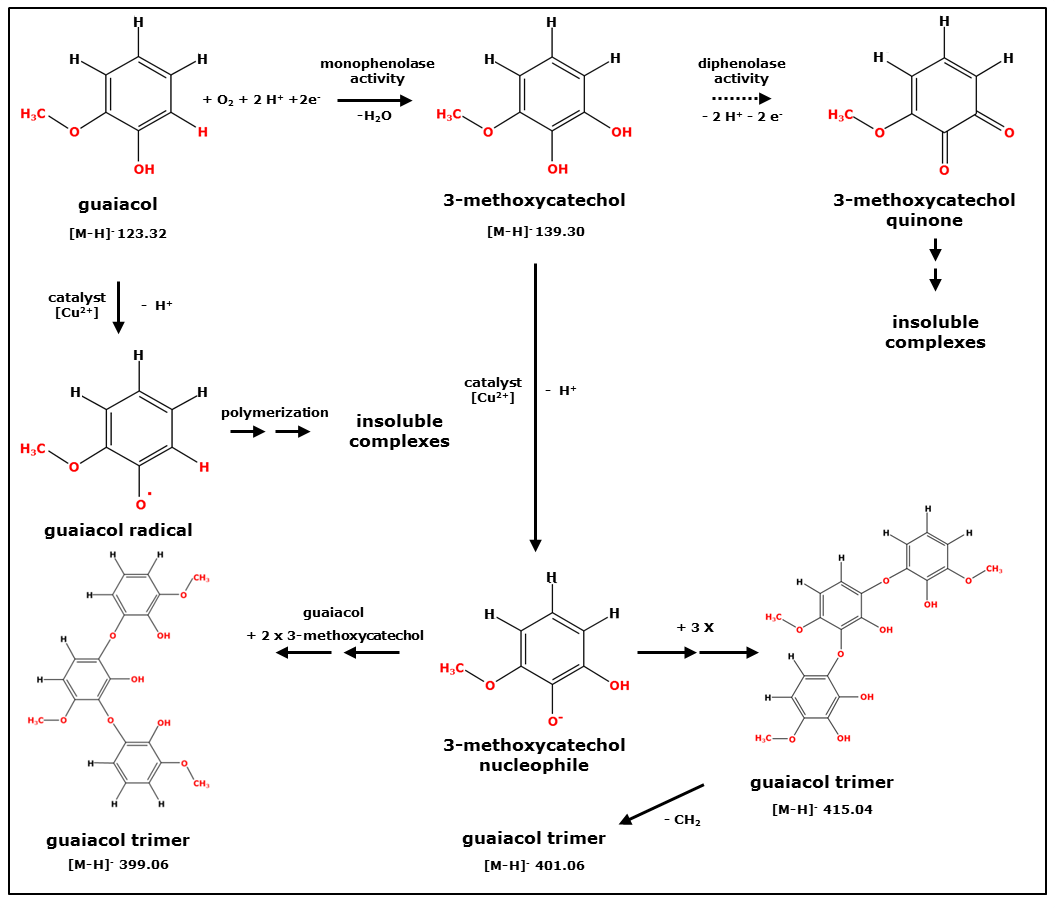

Supplement: Supplementary file 6 — Additional file 6: Figure S5. Schematic presentation of possible reaction pathways of guaiacol incubated in the presence of MtPPO7. In short, MtPPO7 hydroxylates guaiacol (no. 9 specified in Table 1) into 3-methoxycatechol (monophenolase activity). Although not determined, it is likely that 3-methoxycatechol is further oxidized by MtPPO7 into the corresponding ortho-quinone (diphenolase activity, dashed arrow). These ortho-quinones are expected to polymerize and form insoluble complexes. Guaiacol itself forms insoluble complexes via auto-oxidation, which results from the presence of copper during the incubation for 24 h at 50 °C. The decrease in guaiacol concentration during the incubation of guaiacol without MtPPO7 is also shown in Additional file 3: Figure S3a. The determined masses indicate the presence of multiple trimers (399.06, 401.06 and 415.04) consisting of polymerized 3-methylcatechol and guaiacol (Additional file 5: Figure S4). As described above, the polymerization reactions are expected to be catalyzed by copper during the incubation conditions applied. All masses were determined by UHPLC-UV-MSn after incubation of guaiacol (2 mM) with MtPPO7 (5.0 μg mL−1). Samples were incubated for 24 h at 50 °C in 50 mM potassium phosphate, pH 6.0, containing 2.5 µM copper(II)-chloride. [file 13068_2017_810_MOESM6_ESM.png]

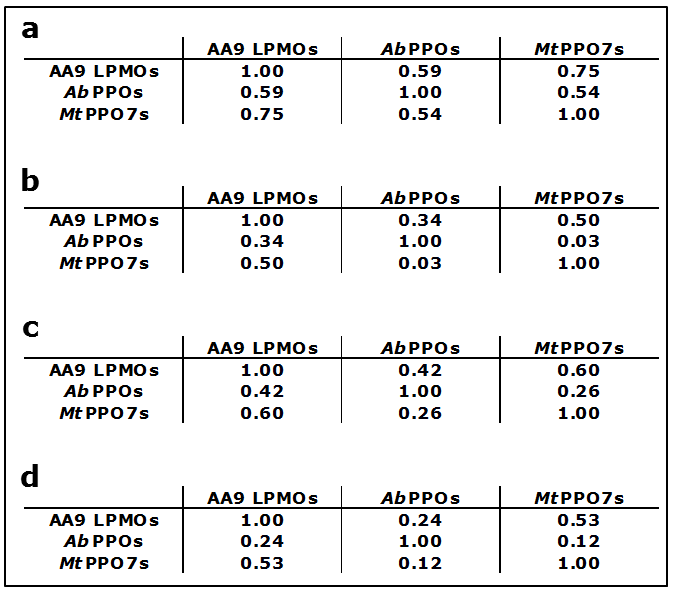

Supplement: Supplementary file 9 — Additional file 9: Figure S6. Correlation of AA9 LPMOs, AbPPOs and MtPPO7s encoding genes in Ascomycota and Basidiomycota. Correlation between the three gene families encoding AA9 LPMOs, AbPPOs and MtPPO7s of (a) 336 Ascomycota and (b) 208 Basidiomycota. Correlation between genes encoding AA9 LPMOs, AbPPOs and MtPPO7s of (c) 27 Ascomycota and (d) 23 Basidiomycota, which have at least ten annotated genes encoding cellulose-degrading enzymes. Species of selected fungal classes are listed in Additional file 7: Table S2 and Additional file 8: Table S3. Graphical presentations of the correlations of (a) together with (b) and (c) and (d) are shown in Fig. 7. See “Methods” for details. [file 13068_2017_810_MOESM9_ESM.png]
