# Supplementary material for: Boosting LPMO-driven lignocellulose degradation by polyphenol oxidase-activated lignin building blocks
Source: Biotechnol Biofuels. 2017 May 10;10:121. doi: 10.1186/s13068-017-0810-4 (PMC5424327; doi:10.1186/s13068-017-0810-4)
Supplement: Supplementary file 7 — Additional file 7: Table S2. Selected cellulase-rich Ascomycota from the JGI database1. [file 13068_2017_810_MOESM7_ESM.docx]

**Additional Table 2** Selected cellulase rich Ascomycota from the JGI database^1^.

| **No** | **Class** | **Fungi** | **Species Description** | **JGI Abbreviation** |
| --- | --- | --- | --- | --- |
| 1  2  3  4  5  6  7  8  9  10  11  12  13  14  15  16  17  18  19  20  21  22  23  24  25  26  27 | Ascomycota  Ascomycota  Ascomycota  Ascomycota  Ascomycota  Ascomycota  Ascomycota  Ascomycota  Ascomycota  Ascomycota  Ascomycota  Ascomycota  Ascomycota  Ascomycota  Ascomycota  Ascomycota  Ascomycota  Ascomycota  Ascomycota  Ascomycota  Ascomycota  Ascomycota  Ascomycota  Ascomycota  Ascomycota  Ascomycota  Ascomycota | *Arthrobotrys oligospora*  *Aspergillus clavatus*  *Aspergillus flavus*  *Aspergillus fumigatus*  *Aspergillus niger*  *Aspergillus oryzae*  *Aspergillus terreus*  *Cladonia grayi*  *Cladosporium fulvum*  *Cochliobolus sativus*  *Debaryomyces hansenii*  *Dothistroma septosporum*  *Fusarium graminearum*  *Fusarium oxysporum*  *Fusarium verticillioides*  *Gaeumannomyces graminis*  *Magnaporthe poae*  *Myceliophthora thermophila*  *Neosartorya fischeri*  *Neurospora crassa*  *Penicillium chrysogenum*  *Pyrenophora teres*  *Sclerotinia sclerotiorum*  *Talaromyces stipitatus*  *Trichoderma reesei*  *Tuber melanosporum*  *Verticillium dahliae* | *Arthrobotrys oligospora* ATCC 24927  *Aspergillus clavatus* NRRL 1 from AspGD  *Aspergillus flavus* NRRL3357  *Aspergillus fumigatus* A1163  *Aspergillus niger* ATCC 1015 v4.0  *Aspergillus oryzae* RIB40  *Aspergillus terreus* NIH 2624  *Cladonia grayi Cgr* DA2myc ss v2.0  *Cladosporium fulvum* v1.0  *Cochliobolus sativus* ND90Pr v1.0  *Debaryomyces hansenii*  *Dothistroma septosporum* NZE10 v1.0  *Fusarium graminearum* v1.0  *Fusarium oxysporum f. sp. lycopersici* 4287 v2  *Fusarium verticillioides* 7600 v2  *Gaeumannomyces graminis var. tritici* R3-111a-1  *Magnaporthiopsis poae* ATCC 64411  *Myceliophthora thermophila/Sporotrichum thermophile* v2.0  *Neosartorya fischeri* NRRL 181  *Neurospora crassa* OR74A v2.0  *Penicillium chrysogenum* v1.0  *Pyrenophora teres f. teres*  *Sclerotinia sclerotiorum* v1.0  *Talaromyces stipitatus* ATCC 10500  *Trichoderma reesei* RUT C-30 v1.0  *Tuber melanosporum* Mel28 v1.0  *Verticillium dahliae* v1.0 | Artol1  Aspcl1  Aspfl1  Aspfu_A1163_1  Aspni7  Aspor1  Aspte1  Clagr3  Clafu1  Cocsa1  Debha1  Dotse1  Fusgr1  Fusox2  Fusve2  Gaegr1  Magpo1  Spoth2  Neofi1  Neucr2  Pench1  Pyrtt1  Sclsc1  Talst1_2  TrireRUTC30_1  Tubme1  Verda1 |

^1^The presented Ascomycota contain at least 10 genes encoding cellulose degrading enzymes, which are classified in the CAZy database as glycosyl hydrolase families GH1, GH3, GH5, GH6, GH7, GH12, GH45. The GH gene families were selected based on Kubicek et al. (2014). The data published by Zhao et al. (2013) was used to determine the amount of annotated genes encoding cellulose degrading enzymes. Based on this selection, a protein BLAST was performed for *Ab*PPOs, AA9 LPMOs and *Mt*PPOs using the protein sequence annotations from the JGI database. The outcome is presented in Figure 7. See M&M for more information.
