# Supplementary material for: Boosting LPMO-driven lignocellulose degradation by polyphenol oxidase-activated lignin building blocks
Source: Biotechnol Biofuels. 2017 May 10;10:121. doi: 10.1186/s13068-017-0810-4 (PMC5424327; doi:10.1186/s13068-017-0810-4)
Supplement: Supplementary file 8 — Additional file 8: Table S3. Selected cellulase-rich Basidiomycota from the JGI database1. [file 13068_2017_810_MOESM8_ESM.docx]

**Additional Table 3** Selected cellulase rich Basidiomycota from the JGI database^1^

| **No** | **Class** | **Fungi** | **JGI Species Description** | **JGI Abbreviation** |
| --- | --- | --- | --- | --- |
| 1  2  3  4  5  6  7  8  9  10  11  12  13  14  15  16  17  18  19  20  21  22  23 | Basidiomycota  Basidiomycota  Basidiomycota  Basidiomycota  Basidiomycota  Basidiomycota  Basidiomycota  Basidiomycota  Basidiomycota  Basidiomycota  Basidiomycota  Basidiomycota  Basidiomycota  Basidiomycota  Basidiomycota  Basidiomycota  Basidiomycota  Basidiomycota  Basidiomycota  Basidiomycota  Basidiomycota  Basidiomycota  Basidiomycota | *Botryobasidium botryosum*  *Coniophora puteana*  *Coprinopsis cinerea*  *Cryptococcus neoformans*  *Dichomitus squalens*  *Fomitiporia mediterranea*  *Fomitopsis pinicola*  *Ganoderma sp.*  *Gloeophyllum trabeum*  *Gymnopus luxurians*  *Hebeloma cylindrosporum*  *Hypholoma sublateritium*  *Jaapia argillacea*  *Laccaria amethystina*  *Laccaria bicolor*  *Melampsora laricis-populina*  *Moniliophthora perniciosa*  *Paxillus involutus*  *Postia placenta*  *Puccinia graminis*  *Puccinia triticina*  *Schizophyllum commune*  *Serpula lacrymans* | *Botryobasidium botryosum* v1.0  *Coniophora puteana* v1.0  *Coprinopsis cinerea* AmutBmut pab1-1 v1.0  *Cryptococcus neoformans var neoformans* JEC21  *Dichomitus squalens* v1.0  *Fomitiporia mediterranea* v1.0  *Fomitopsis pinicola* FP-58527 SS1 v3.0  *Ganoderma sp.* 10597 SS1 v1.0  *Gloeophyllum trabeum* v1.0  *Gymnopus luxurians* v1.0  *Hebeloma cylindrosporum* h7 v2.0  *Hypholoma sublateritium* v1.0  *Jaapia argillacea* v1.0  *Laccaria amethystina* LaAM-08-1 v2.0  *Laccaria bicolor* v2.0  *Melampsora laricis-populina* v2.0  *Moniliophthora perniciosa* FA553  *Paxillus involutus* ATCC 200175 v1.0  *Postia placenta* MAD-698-R-SB12 v1.0  *Puccinia graminis f. sp. tritici* v2.0  *Puccinia triticina* 1-1 BBBD Race 1  *Schizophyllum commune* H4-8 v3.0  *Serpula lacrymans* S7.9 v2.0 | Botbo1  Conpu1  Copci_AmutBmut1  Cryne_JEC21_1  Dicsq1  Fomme1  Fompi3  Gansp1  Glotr1_1  Gymlu1  Hebcy2  Hypsu1  Jaaar1  Lacam2  Lacbi2  Mellp2_3  Monpe1_1  Paxin1  PosplRSB12_1  Pucgr2  Puctr1  Schco3  SerlaS7_9_2 |

^1^The presented Basidiomycota contain at least 10 genes encoding cellulose degrading enzymes, which are classified in the CAZy database as glycosyl hydrolase families GH1, GH3, GH5, GH6, GH7, GH12, GH45. The GH gene families were selected based on Kubicek et al. (2014). The data published by Zhao et al. (2013) was used to determine the amount of annotated genes encoding cellulose degrading enzymes. Based on this selection, a protein BLAST was performed for *Ab*PPOs, AA9 LPMOs and *Mt*PPOs using the protein sequence annotations from the JGI database. The outcome is presented in Figure 7. See M&M for more information.
